# MB-GAN: Microbiome Simulation via Generative Adversarial Network

**DOI:** 10.1093/gigascience/giab005

**Published:** 2021-02-05

**Authors:** Ruichen Rong, Shuang Jiang, Lin Xu, Guanghua Xiao, Yang Xie, Dajiang J Liu, Qiwei Li, Xiaowei Zhan

**Affiliations:** University of Texas Southwestern Medical Center, Quantitative Biomedical Research Center, Department of Population and Data Sciences, 5323 Harry Hines Blvd, Dallas, TX 75390, USA; University of Texas Southwestern Medical Center, Quantitative Biomedical Research Center, Department of Population and Data Sciences, 5323 Harry Hines Blvd, Dallas, TX 75390, USA; Southern Methodist University, Department of Statistical Science, 3225 Daniel Ave, Dallas, TX 75275, USA; University of Texas Southwestern Medical Center, Quantitative Biomedical Research Center, Department of Population and Data Sciences, 5323 Harry Hines Blvd, Dallas, TX 75390, USA; University of Texas Southwestern Medical Center, Quantitative Biomedical Research Center, Department of Population and Data Sciences, 5323 Harry Hines Blvd, Dallas, TX 75390, USA; University of Texas Southwestern Medical Center, Quantitative Biomedical Research Center, Department of Population and Data Sciences, 5323 Harry Hines Blvd, Dallas, TX 75390, USA; Pennsylvania State University, Department of Public Health Sciences, 700 HMC Crescent Road, Hershey, PA 17033, USA; University of Texas at Dallas, Department of Mathematical Sciences, FN32 800 West Campbell Road, Richardson, TX 75080, USA; University of Texas Southwestern Medical Center, Quantitative Biomedical Research Center, Department of Population and Data Sciences, 5323 Harry Hines Blvd, Dallas, TX 75390, USA; University of Texas Southwestern Medical Center, 5323 Harry Hines Blvd, Dallas, TX 75390, USA. Center for the Genetics of Host Defense

**Keywords:** microbiome simulation, generative adversarial network, deep learning

## Abstract

**Background:**

Trillions of microbes inhabit the human body and have a profound effect on human health. The recent development of metagenome-wide association studies and other quantitative analysis methods accelerate the discovery of the associations between human microbiome and diseases. To assess the strengths and limitations of these analytical tools, simulating realistic microbiome datasets is critically important. However, simulating the real microbiome data is challenging because it is difficult to model their correlation structure using explicit statistical models.

**Results:**

To address the challenge of simulating realistic microbiome data, we designed a novel simulation framework termed MB-GAN, by using a generative adversarial network (GAN) and utilizing methodology advancements from the deep learning community. MB-GAN can automatically learn from given microbial abundances and compute simulated abundances that are indistinguishable from them. In practice, MB-GAN showed the following advantages. First, MB-GAN avoids explicit statistical modeling assumptions, and it only requires real datasets as inputs. Second, unlike the traditional GANs, MB-GAN is easily applicable and can converge efficiently.

**Conclusions:**

By applying MB-GAN to a case-control gut microbiome study of 396 samples, we demonstrated that the simulated data and the original data had similar first-order and second-order properties, including sparsity, diversities, and taxa-taxa correlations. These advantages are suitable for further microbiome methodology development where high-fidelity microbiome data are needed.

## Background

The microbiome is a collection of trillions of microorganisms living within humans. Previous studies have revealed that the microbiome has a profound impact on human disease, including inflammatory bowel disease, colorectal cancer, Type 2 diabetes, and psychiatric disorders [1–4]. A powerful and increasingly popular method to study the microbiome and disease is metagenome-wide association studies (MWAS). These studies use the taxonomic abundance data of thousands of bacteria generated from sequencing instruments and calculate the association strengths between the bacterial abundances and the phenotypes. As researchers have expanded their interests into studying microbial associations in human physiology, MWAS has taken on an increasingly critical role.

A successful MWAS relies on valid statistical models [5], and the evaluation of MWAS models relies on simulations. As for method development, designing benchmark settings that fully capture the characteristics of the microbiome data could reasonably reflect the quality across various models. For example, Jiang et al. [6] demonstrated that edgeR, a statistical model proposed for analyzing RNA-seq data [7], showed inferior performance on data with a much higher sparsity such as microbiome data. Furthermore, a reasonable summary of the model performance based on simulation guides real-world applications. In practice, the user can specify realistic scenarios in simulation and select the best model with the highest empirical statistical power.

Although simulation plays a vital role in MWAS, it is not trivial to simulate microbiome abundances with high fidelity to the real data. Microbiome abundances are sparse, overdispersed (large variances compared to means), and have an intrinsic phylogenetic relationship [8]. In addition, as the microbiome consists of interactive communities, the microbiota form complex taxa-taxa relationships with a nonnegligible second-order covariation [9,10]. However, a simulation method that can capture the first-order (e.g., sample-level) characteristics while maintaining the second-order (e.g., taxa-taxa level) relationships is lacking in the current literature. For example, explicit statistical distributional assumptions, such as the zero-inflated logistic normal distribution, were introduced to simulate individual bacterial taxa [11]. Although the simulated data have the desired sample-level and taxon-level properties (e.g., sparsity and overdispersion), they ignore the taxa-taxa relationships. Other methods, such as Normal-to-Anything (NorTA) [12], have attempted to model the taxa-taxa relationships, but their performance at the sample level is not satisfactory, as we demonstrate later in this article.

Given the aforementioned challenges in explicitly modeling microbiome abundances, we developed a novel deep learning–based approach to implicitly compute simulated microbial abundances with desired sample-level and taxa-taxa interactive characteristics. We refer to this simulation framework as MB-GAN (Microbiome Generative Adversarial Network) because it is adapted from a generative adversarial network (GAN) framework [13] and is customized for simulating microbiome datasets. GAN has been a feature of deep learning studies since its foundational work proposed in 2014 [13]. It has a generator network and a discriminator network. The generator network takes random noise and outputs simulated data. The discriminator network takes both the simulated and the real datasets and distinguish the inputs as real or fake. In the network training stage, the generator focuses on increasing the similarity between the simulated data and the real data; meanwhile, the discriminator works on better distinguishing between the simulated and the real data. When the training stage finishes, the generator will be able to simulate data that are hard for the discriminator to distinguish from the real data. Owing to the excellent performance of GANs compared with conventional approaches (e.g., variational auto-encoder [14]), GAN-based models have wide applications, such as image synthesis (e.g., human facial images [15]), text generation (e.g., visual paragraph generation [16]) and music synthesis (e.g., music composition [17]). Recently, GAN models have also been adapted for biomedical research. For example, GAN models have been applied in generating sequence data (e.g., T-cell receptor sequences [18]) and enhancing medical imaging [19]. Given the lack of performant simulation models for microbiome datasets and the impressive potential of GAN-based simulation models, we are motivated to incorporate the GAN model to simulate microbiome abundances.

In this article we contribute a novel microbiome simulation model, MB-GAN, and show that it can simulate high-fidelity microbiome abundances. Specifically, we modified the discriminator network to incorporate microbiome diversity-based measurements. Compared with the original GAN framework, our algorithm converges fast and robustly. It can thus easily be applied to simulate new datasets based on a set of input microbiome abundances without explicit modeling. In a real data study, we demonstrate that the simulated microbiome abundances have similar data characteristics, including both first-order (sample-level properties such as sparsity and diversity) and the second-order properties (taxa-taxa correlations). Thus the simulated data can be used in further methodology development and evaluations.

## Data Description

We benchmarked the MB-GAN model using a real sequencing dataset from a human gut microbiome study published by Nielsen et al. [20]. The dataset contains 396 sequenced shotgun metagenomic samples from 148 patients with inflammatory bowel disease and 248 healthy controls. The original sequencing data from the fecal samples are available in the European Nucleotide Archive (ENA) database with the study number PRJEB1220. We used curatedMetagenomicData [21] to obtain a taxonomic abundance table of all samples with 1,939 detected taxa at different taxonomic levels. We further separated the samples into case (patients with inflammatory bowel disease) and control (healthy controls) groups to implement MB-GAN separately (see more details in Methods section). We also used the phylogenetic tree that accompanied the original sequencing data provided by curatedMetagenomicData. In addition, results from MB-GAN on a microbiome study with smaller sample sizes (111 in total) are available in section “Evaluating MB-GAN on a Smaller Microbiome Dataset” in the Supplement.

## Analyses

To simulate microbiome abundances using MB-GAN, we used the 148 cases (or 248 controls) as real data input to train MB-GAN. We set 100,000 iterations with a batch size of 32 (see details in Methods section). We simulated 1,000 MB-GAN samples for each group separately, and both the generators and critics reached convergence in 10 minutes after 20,000 iterations. For a brief illustration of how similar the MB-GAN samples were to the real ones, we picked the 60 most abundant taxa from the 148 case samples and compared their abundances between the real and simulated data. As a comparison, we also considered 2 additional simulation methods: NorTA and metaSPARSim [12,22]. NorTA was designed to generate multivariate random variables with a prespecified correlation structure, and metaSPARSim is a model-based approach to simulate 16S ribosomal RNA sequencing count data. More details are provided in the Methods section. Briefly speaking, we applied NorTA to the same real dataset, where we generated 1,000 samples based on the 148 case and 248 control samples, respectively. Similarly, we used metaSPARSim to simulate microbiome abundances of the same sample sizes. By comparing the performances of MB-GAN and other methods, we demonstrated the high fidelity of MB-GAN samples to the real data (Supplementary Fig. S1a and b), as there are similar patterns of abundances shared between the real datasets and MB-GAN simulated datasets. We further compared the simulated and observed abundances to examine the fidelity of the MB-GAN samples in different taxa abundance strata. In the case group, we considered (1) the taxa having <$10\%$ zeros across all the samples and (2) the taxa having 10–20% zeros across all the samples. In each scenario, we compared the observed and simulated abundances (by MB-GAN and other methods) using the Wilcoxon rank-sum test. The MB-GAN samples gave *P*-values >0.05 in both scenarios, whereas the NorTA and metaSPARSim samples showed significantly different abundances (both *P*-values <0.0001) when compared with the observed data for those abundant taxa (Supplementary Fig. S2). We performed the same analysis on the control group. The *P*-values are 0.89 for scenario 1 and 0.031 for Scenario 2 when testing the MB-GAN simulated abundances against the observed ones. Again, the *P*-values for the NorTA and metaSPARSim results were <0.0001 (Supplementary Fig. S3). This showed that MB-GAN was well able to simulate the highly abundant taxa in the real data. In contrast, the NorTA simulated abundances are smaller in magnitude compared with both the observed and the MB-GAN abundances, and metaSPARSim did not perform well either. We concluded that neither NorTA nor metaSPAR shared a good pattern with the observed abundances. We performed the same analysis on the smaller sample size dataset and reached the same conclusions. As shown in Supplementary Fig. S4, the Wilcoxon rank-sum test for the MB-GAN samples yielded *P*-values >0.05 for the case and control groups, while the *P*-values for the NorTA and metaSPARSim results were <0.0001.

### Evaluation on sample-level properties

First, we evaluated sample sparsity, which is the proportion of zeros in a sample. For the real data, the observed sparsity ranges from 0.71 to 0.90, with median values being 0.80 and 0.83 for the case and the control group, respectively. As for the 2 types of simulated data, the lower bound of sample sparsity by MB-GAN matched well with the real data for both groups, but in general the MB-GAN simulated data showed a slightly higher sparsity, with a median of 0.83 and 0.85 for the case and the control group, respectively. NorTA simulated data, on the other hand, tended to underestimate the sparsity in both case and control groups. The median values of the sample sparsity were both <0.80 for the 2 groups, and the maximum sparsity was <0.85. The sparsity given by metaSPARSim was also lower than the actual values, in general. The overall sparsity by metaSPARSim ranged from 0.71 to 0.84, with the median being 0.77 and 0.80 for the case and the control group, respectively. The maximum sparsity was only 0.84, which was smaller than the observed maximum (0.90) from the real data. Thus, MB-GAN simulated data could better capture the sparsity observed across all the samples of the real data. This conclusion also held for the smaller sample size case, as reported in Supplementary Table S1.

**Figure 1:**
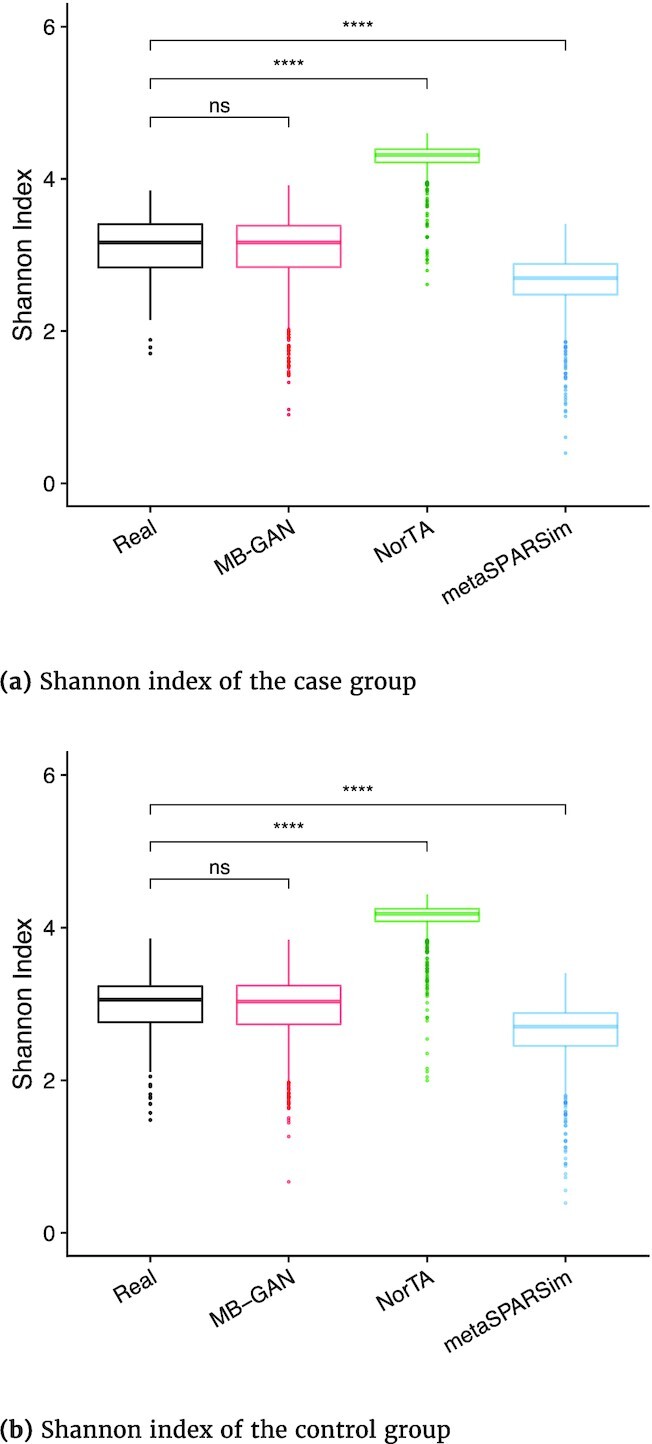
Box plots of Shannon index calculated from real data and 3 simulated datasets (MB-GAN, Normal-To-Anything [NorTA], and metaSPARSim). Notations: ns – not significant; **** – p-value < 0.0001.

Next, we evaluated the α-diversities of the simulated data from MB-GAN, NorTA, and metaSPARSim. We compared the Shannon indices calculated from the simulated and the real samples. The Shannon index is a metric that weights the relative abundance of species by their relative evenness in a sample (see details in Methods section). As an α-diversity index, it provides more information than simply species richness (i.e., the number of species in the sample) because it considers the relative abundances of different species. Therefore, a better match between the Shannon indices calculated from the simulated data and the real data suggested that the simulator could better characterize the biodiversity of the real data. As shown by the box plots in Fig. 1a and b, the Shannon indices calculated from MB-GAN simulation data matched consistently with the real data in both the case and the control groups. The *P*-value given by the Wilcoxon rank-sum test was 0.83 for the case group and 0.57 for the control group. As for the 2 alternative methods, the Shannon indices by NorTA were larger than the real ones, and the variation among the results was not well characterized. This suggests that the samples simulated by NorTA were less diverse compared to the real data. In contrast, the Shannon indices by metaSPARSim were smaller, suggesting a higher diversity in the metaSPARSim samples than the real data. Again, the Wilcoxon rank-sum test yielded *P*-values <0.0001 for both groups when comparing the real data against NorTA or metaSPARSim simulated data. The box plots in Supplementary Fig. S5 for the smaller sample size case also demonstrated the same conclusion. In all, our results suggested that the MB-GAN simulated data better resembled the real microbiome abundances with respect to α-diversity.

**Figure 2:**
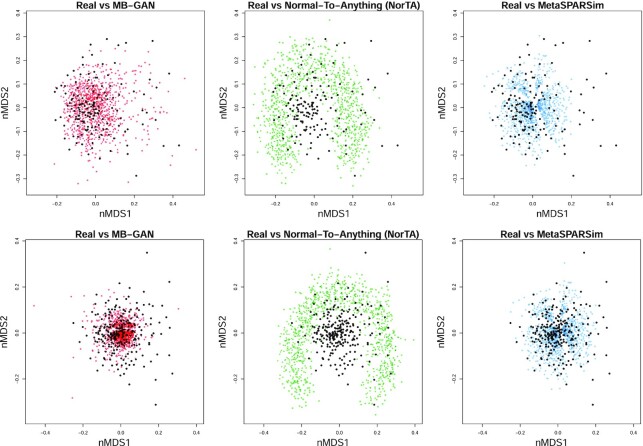
β-diversity visualization using non-metric multidimensional scaling (nMDS) for (a) case and (b) control groups. For samples from the real data, MB-GAN, Normal-To-Anything (NorTA), and metaSPARSim simulated data, the 2D nMDS values were calculated using the unweighted UniFrac metric.

Finally, we calculated the β-diversity of the simulated data from MB-GAN, NorTA, and metaSPARSim and compared the results with the β-diversity of the real data. β-diversity measures how different samples are from each other (see more details in Methods section), and a commonly used way to visualize β-diversity is by non-metric multidimensional scaling (nMDS) analysis. Here, the samples were compared on the basis of their species-level abundances. We incorporated the species’ phylogenetic information by using UniFrac distance [23] matrix to generate the nMDS plots. Figure 2a and b visualizes the results from the case and the control group, respectively. In each panel, the black dots represent the results from the real data, and different colored dots represent the results from 3 simulations. For both groups, the clear overlap between the real data and the MB-GAN simulated data demonstrated the similarity between those samples. The NorTA simulated data, however, showed a unique circular pattern that was different from either the real data or the MB-GAN simulated data. The results given by metaSPARSim were less spread out than the real data. It can also be seen that the blue points form smaller groups separated by gaps. Note that these gaps are not observed for the real data. Interestingly, the aforementioned patterns were also observed for the smaller sample size case, as shown in Supplementary Fig. S6. Compared to the 2 alternative methods, data generated by MB-GAN demonstrate a more reasonable representation of the real data in terms of the UniFrac β-diversity.

### Evaluation on taxa-taxa relationships

In addition to comparing the sample-level similarities, we illustrated that MB-GAN had the advantage of preserving the second-order characteristics in the real data. We measured the taxa-taxa relationships using (i) Spearman correlation coefficients and (ii) proportionality between taxa pairs. We visualized the correlation matrices and the empirical distributions of the correlation coefficients to compare the real data and the 3 types of simulated data (MB-GAN, NorTA, and metaSPARSim). Here, the comparison considered the top 10% most abundant species in the real data, because these taxa contained more information in capturing the taxa-taxa interactions. The names of the remaining species can be found in Supplementary Figs S7 and S8 for the case and the control group, respectively.

**Figure 3:**
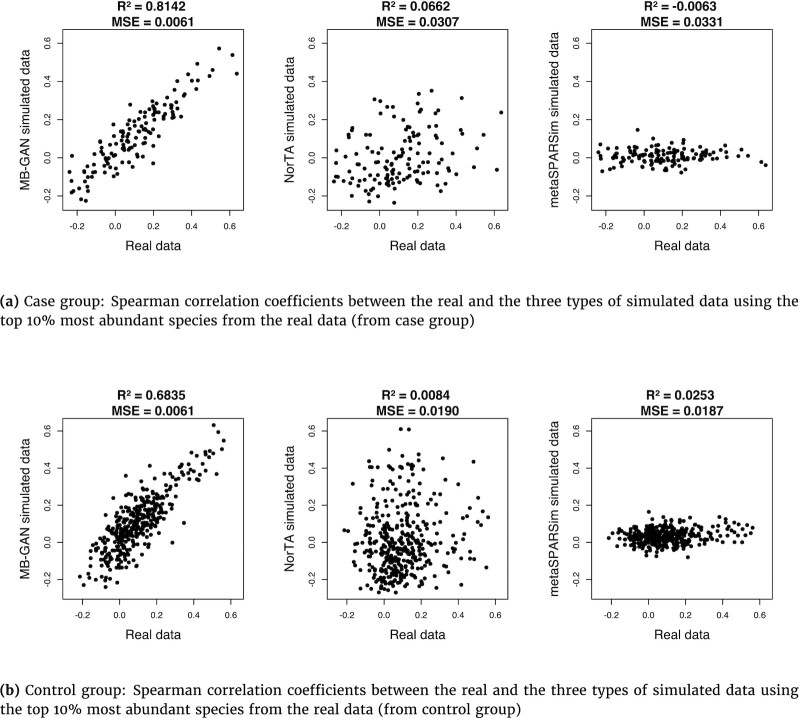
Scatter plot of the Spearman correlation coefficients between the real and the simulated data by MB-GAN, Normal-To-Anything (NorTA), and metaSPARSim. Results are calculated based on the top 10% most abundant species from the real data of the (a) case and the (b) control group.

The scatter plots in Fig. 3a and b visualize the Spearman correlations calculated from the real data against the simulated data. The *R*^2^ and mean square error (MSE) are included in the plot. In general, there was a relatively strong linear trend in the first panel of Fig. 3a and b, suggesting that the correlation structure from MB-GAN samples resembled that of the real data for both groups. In contrast, for the other 2 simulators, the Spearman correlations seemed to be quite different from the real data, as shown in the second and third panels in Fig. 3a and b. The results given by metaSPARSim suggest that the simulated taxa had weak association measured by the Spearman correlation coefficient. As for the smaller sample size case, Supplementary Fig. S9 also demonstrates that MB-GAN samples better preserved the correlation structure in the real data. Supplementary Figs S10a and S11a compare the patterns in correlograms of Spearman correlation matrices, calculated from the real and simulated data. A blue ellipse represents a negative correlation, while a red one suggests a positive correlation. The darker the color or the shorter the ellipse’s minor axis, the stronger the correlation between the corresponding taxa pair. MB-GAN was able to capture the overall pattern of the correlogram from the real data, and it preserved the relatively strong associations for both the case and the control groups. The same conclusion held for the smaller sample size case, as shown in Supplementary Fig. S12. However, all 3 simulators tended to show weaker associations compared to the real data, especially for metaSPARSim. Furthermore, we observed a clear disparity between the correlograms by NorTA and the real data. Supplementary Figures S10b and S11b overlay the empirical distributions of the Spearman correlation coefficients from the real data and a type of simulated data. For both the case and the control group, the empirical distributions given by MB-GAN better matched the true coefficients’ distributions. In all, Fig. 3 and Supplementary Figs S10 and S11 illustrate the overall better performance of MB-GAN over NorTA and metaSPARSim with respect to capturing the taxa-taxa relationships measured by Spearman correlation.

In addition to the Spearman correction, we further compared the taxa-taxa relationships using the idea of “proportionality” proposed by Lovell et al. [24]. Proportionality was designed to analyze relative data. It has the advantage of preserving the associations between a pair of abundances for taxon *m* and *k* across all samples, (*x_m_*, *x_k_*), in their absolute scale. We calculated the “goodness-of-fit to proportionality” statistic ϕ as ϕ(log *x_m_*, log *x_k_*) = var(log (*x_m_*/*m_k_*))/var(log *x_m_*), as suggested by Lovell et al. [24]. Furthermore, it is recommended to apply the centered logratio (clr) transformation on the sample level to ensure that the results between different variable pairs are comparable. Specifically, $\mathrm{clr}(x_m) = \log [x_m/g (x)]$, with *g*(*x*) being the geometric mean of the sample *x*.

**Figure 4:**
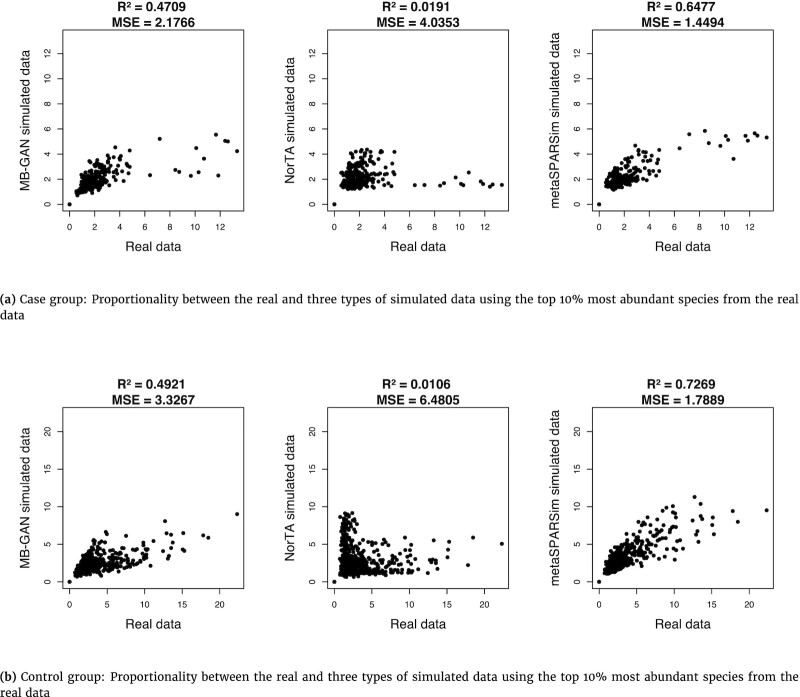
Scatter plot of the “goodness-of-fit proportionality” statistic between the real and the simulated data by MB-GAN, Normal-To-Anything (NorTA), and metaSPARSim. Results are calculated based on the top 10% most abundant species from the real data of the (a) case and the (b) control group.

We presented the results using scatter plots to examine Lovell proportionalities for the case group and the control group for all simulation methods (Fig. 4). We also calculated the correlation of coefficients (*R*^2^) and MSEs based on all points in each panel. We found higher *R*^2^ values and lower MSEs for MB-GAN and metaSPARSim compared with NorTA. Additionally, based on the heat maps in Supplementary Fig. S13, we observed that both MB-GAN and metaSPARSim shared a lot of similarities with the heat maps of real data for both the case and the control groups. These observations also held when we used a smaller sample size (Supplementary Fig. S14). In general, MB-GAN and metaSPARSim performed reasonably well in preserving the proportionality of real data. Notably, as shown in the previous section, MB-GAN could outperform metaSPARSim by preserving sample-level properties of the real data.

## Discussion

While MWAS greatly facilitate the investigation of association between the human microbiome and diseases, the evaluation of existing MWAS models requires realistic simulation studies. However, it is challenging to specify explicit statistical distributions in simulation to fully mimic the complex patterns observed in real microbiome data. Specifically, few simulation frameworks achieve good performance in both modeling the sample-level characteristics (e.g., sparsity, α-diversity, and β-diversity) and maintaining realistic levels of taxa-taxa associations. To address these challenges, we have developed a novel simulation framework, MB-GAN. It is designed on the basis of the latest research into GANs and adapts phylogenetic transformation and ecologically meaningful discriminator loss for improved convergence. The simulator is trained on real data and does not need explicit statistical models. It can simulate microbiome relative abundances that are not easily distinguishable from real data in terms of sample-level characteristics and taxa-taxa relationships. For example, MB-GAN can mimic original data and provide similar simulated relative abundances in terms of diversity, sparsity, and feature correlations. If a small dataset is not sufficient to train a converged MB-GAN model, we recommend checking the data quality and network structure first and then exploring adaptive learning strategies to gradually add new samples into the model until newly generated data could represent the original training data.

Recently, GAN has been widely adapted to generate simulated data that are hard to distinguish from real data. Researchers have successfully applied GANs to different fields, including text generation, music synthesi, and image synthesis. GANs have also been adapted for biomedical research tasks such as generating sequence data. Our proposed method, MB-GAN, is to our knowledge the first simulation framework that adapts GANs to generate microbiome abundances. Unlike the traditional GANs, our algorithm is easily applicable and can converge efficiently.

However, MB-GAN should not be used as a tool to enlarge existing sample sizes in MWAS. For example, detecting differentially abundant taxa based on MB-GAN enlarged samples may not yield valid biological conclusions. We performed a simple differential abundance analysis in the Supplement (Section “Differential Abundance Analysis by MB-GAN”). The results shown in Supplementary Fig. S15 suggested that it may be invalid to draw biologically meaningful conclusions based on the mixed data consisting of both the MB-GAN simulated samples and the real samples. Alternatively, we recommend using MB-GAN to design the simulation study and evaluate MWAS models. These models can be critically important to understand the relationship between microbiome and health in the future.

## Potential Implications

MB-GAN can benefit future quantitative methodology development for microbiome research. For example, MB-GAN simulated data can be used to evaluate statistical models designed for MWAS studies: researchers can use MB-GAN to simulate microbiome abundances for a certain sample size and impose the statistical effect sizes on a subset of taxa for different phenotype groups [25], perform analysis using these MWAS models, and classify the detected differentially abundant taxa into true-positive and false-positive results. In this way, researchers can examine the Type I error (false positive) and power of these MWAS models. A concrete example of using an MB-GAN sample to evaluate MiRKAT [25], a widely used statistical model proposed for MWAS, is available in section “Using MB-GAN to Design a Simulation Study to Evaluate MiRKAT” in the Supplement. Supplementary Fig. S16 summarized the Type I error and statistical power of MiRKAT based on the simulation designed by using an MB-GAN sample. In addition, investigations of microbiome networks can also use MB-GAN because MB-GAN can effectively preserve the taxa-taxa interactions compared to other existing approaches. Statistical models that focus on detecting the correlation-based microbiome co-occurrence pattern can use the MB-GAN samples for model evaluation. In conclusion, MB-GAN enables the evaluation of various types of microbiome studies by providing simulated data with high fidelity to the real data.

## Methods

### Construction of MB-GAN

We created MB-GAN using a generator network and a discriminator network, which resembled the classic GAN network. The goal of the generator network is to take random noise, conduct a series of non-linear transformations, and compute simulated microbiome relative abundances. The goal of the discriminator is to distinguish whether the data are from the generator or from the real datasets. While the MB-GAN architecture was similar to the GAN framework, we designed 2 specific layers to make it more usable for microbiome data. First, we added a phylogenetic transformation layer. Because many microbial taxa have low abundances, we can transform them to larger values to achieve better model convergence. Second, we used Earth Mover’s (EM) distance to compute the loss function for the discriminator network. This can quantitatively measure the similarity between any 2 microbiome samples given their phylogenetic information. The computed diversity value was used as the loss value in the discriminator network.

The usability of the MB-GAN network was based on the Wasserstein GAN with gradient penalty (WGAN-GP) framework [26], which exhibits several improvements over the classic GAN framework [13]. Briefly speaking, the classic GAN has 2 major drawbacks: (i) the generator stops updating when the discriminator is overpowered (e.g., the generator’s gradients vanish or explode) and (ii) the generated samples lack enough variability (model collapse [27]). To overcome these problems and fully unleash the power of GAN, WGAN was proposed [28]. It invented a scalar similarity score (e.g., the EM distance) as a critic to measure the quality of the simulated data, and replaced the original binary discriminator that distinguished whether the data are real or fake by probabilities. In both theory and practice, WGAN provided a smoother gradient everywhere and thus the generator continued to learn new knowledge even when the critic already performed well. Therefore, we used a scalar loss value computed by the EM distance in the MB-GAN discriminator network. The EM distance was introduced in WGAN [28], so MB-GAN shared the same benefits as those from WGAN. To further improve the model convergence and simulation quality, we incorporated the training strategy proposed in WGAN-GP [26], which reformed the gradient clipping into a gradient penalty to solve a practical issue caused by WGAN’s Lipschitz constraint. In our observation, training MB-GAN using this strategy showed no sign of any gradient problems or model collapse and thus was an optimized framework for training a well-performed GAN generator for simulation.

**Figure 5:**
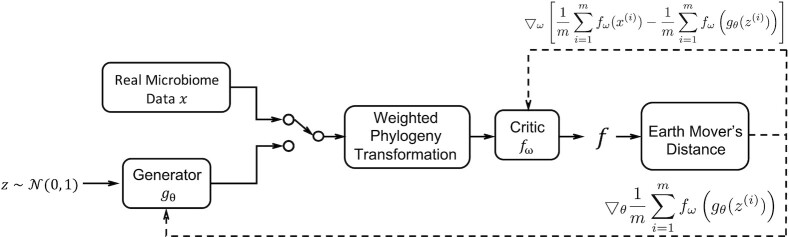
The MB-GAN network architecture. Solid lines: the Gaussian noise *z* is passed to the generator model *g*_θ_; both real microbiome data *x* and simulated microbiome data *g*_θ_(*z*) undergo weighted phylogeny transformation and are passed to the critic model *f*_ω_; the function *f* calculates the Earth Mover’s distance. Dashed lines: the differentiated scores are used to update generator weights θ and critic weights ω at the *i*th iteration.

The detailed architecture of our proposed MB-GAN network structure is illustrated in Fig. 5. In the training phase, the MB-GAN network requires a real microbiome dataset *x*, which contains the relative abundances of taxa observed across different samples. For each sample, the relative abundance of a taxon is defined as its percent composition relative to the total abundances observed in that sample. The generator takes random noise inputs (e.g., $z \sim \mathcal {N}(0, 1)$ as Gaussian noise) and outputs simulated microbiome datasets (*g*_θ_(*z*)). Both real microbiome samples and simulated samples are combined in 1 batch. Then all data would undergo the weighted phylogeny transformation and be sent to a critic to calculate the EM distance between the real and simulated samples. The generator and critic are differentiated against the distance alternately to update the model weights.

To handle microbiome data and incorporate their underlying tree structure, we added a transformation layer. It integrates additional taxonomic information into the critic to biologically measure the dissimilarity between the real sample and the generated sample. First, the transformation layer expands the species-level table based on the hierarchical tree structure to make a full abundance matrix (e.g., aggregate all taxa abundances from phylum to species). When feeding the transformed matrix into the WGAN-GP framework, the critic calculates an EM distance. Notably, the EM distance here is often termed the Wasserstein-1 distance or Kantorovich-Rubinstein metric in the machine learning community [28], and it is not to be confused with the phylogenetic Kantorovich-Rubinstein metric [29]. Second, we can integrate algorithmic transformations into the transformation layer. For example, we can apply logarithmic transformation as a normalization step to our MB-GAN framework. In this article, we transformed each observed relative abundance *x* into $\log [(1+1,000x)/(1+x)]$. In practice, we observed that this transformation provided robust and satisfactory results regarding the first- and second-order properties, even without the help of tree branch length. Supplementary Figure S17 shows how the transformation amplifies the observed small abundances.

### Implementation

#### Data processing

As illustrated in Fig. 5 and Supplementary Fig. S18, the input of the generator model is a Gaussian random noise vector, and the inputs of the critic model are the real data and the simulated data. Next, both the real and the simulated data go through the weighted phylogeny transformation, which uses a rooted taxonomic tree (or phylogenetic tree) to expand the species- (or operational taxonomic unit–) level abundance matrices. The outputs include the abundance of all internal nodes for each input sample. Last, the critic uses the outputs to calculate an EM distance.

#### MB-GAN training

We incorporated the training method in WGAN-GP with our own customization. For the critic, we use Wasserstein loss to measure the EM distance between the real data and the simulated data. Meanwhile, we used the GP loss to achieve the Lipschitz constraint required by WGAN-GP. We weighted the Wasserstein loss and GP loss by 1:10. For the generator, we used Wasserstein loss to measure the minimum cost to change the simulated data into real data. In the MB-GAN training phase, we used RMSprop optimizer with learning rate 5 × 10^−5^ to update the model weights. The generator and critic are differentiated alternately, with 5 steps of critic followed by 1 step of generator in each iteration.

We assessed the convergence of the MB-GAN using 2 statistics. The first statistic combined the Wasserstein loss of generator and the Wasserstein loss of critic. Indeed, these losses can also be used for model selection. The second statistic was the mean of the MSE of the change of model parameters across iterations. The training of critic and generator was considered to reach convergence when the difference of the mean MSE was <1 × 10^−8^ in a 1,000-iteration window. In practice, it is not necessary to stop the model training when the mean MSE is small. As the MB-GAN model shows no sign of collapse during the training, it is recommended to train the model as long as possible even when the model reaches convergence, and then the best model can be selected by comparing model performance.

### Comparison and evaluation of the MB-GAN

#### Comparison with Normal-to-Anything

We compared the MB-GAN to NorTA, an alternative simulation method for microbiome data. NorTA was designed to generate multivariate random variables with a prespecified correlation structure [12]. In general, NorTA transforms a multivariate Gaussian random variable with a given correlation structure to an arbitrary discrete or continuous random variable, where the transformation is determined by the marginal distribution of the target random variable. In recent microbiome network studies, NorTA has been used to generate high-dimensional sparse count data with the underlying correlation structure defined by a target correlation matrix [30,31]. Choosing the zero-inflated negative binomial (ziNB) as the target marginal distribution in the transformation step was shown to well characterize the overdispersion and zero inflation observed in real microbiome data  [30]. Briefly, the NorTA method includes the following steps: (i) remove the taxa with zeros across all real data samples; (ii) generate an *n* × *p* multivariate normal random variable with zero mean and *p* × *p* correlation matrix calculated from the real data, where *n* and *p* are the sample size and taxa number, respectively; (iii) for each taxon *j*, apply a standard normal cumulative distribution function transformation to get a uniform random variable; (iv) apply the quantile function of a ziNB distribution to each uniform random variable to generate the count vector for each taxon *j*, with the parameters of the ziNB distribution estimated from the observed count data of taxon *j*; and (v) compositionalize the resulting *n* × *p* count matrix by dividing each sample (row) by the total counts in that sample. The above steps are implemented in the function synth_comm_from_counts in the R package SPIEC-EASI [30].

#### Comparison with metaSPARSim

metaSPARSim is a 16S rDNA-seq data simulator [22]. It simulates count matrices resembling real sequencing count data using a model-based approach. metaSPARSim consists of 2 steps. The first step models the variation of species abundances between biological replicates through a Gamma distribution. The second step employs a multivariate hypergeometric model to capture the technical variability in the sequencing process. Patuzzi et al. [22] suggested that metaSPARSim was able to generate synthetic data resembling real 16S rDNA-seq data with respect to the compositionality and sparsity. The count matrices given by metaSPARSim could potentially serve for assessing the analytical tools for count data normalization and differential abundance analysis. In this article, we used the functions estimate_parameter_from_data and metaSPARSim in the R package metaSPARSim(version 1.1.1) to implement metaSPARSim. In total, 1,000 samples were generated for the case and the control group, respectively.

#### Evaluation of model performance

To evaluate the quality of the simulated datasets, we compared sample-level statistics and taxa-taxa correlations calculated from the real data and the 2 types of simulated data.

We used sparsity and diversity to measure the sample-level characteristics. The sparsity was defined as the proportion of zeros in a sample. In the MB-GAN outputs, abundances <1 × 10^−4^ were truncated to zero. The α-diversity for each sample was defined as the Shannon index: $-\sum _{j = 1}^p p_{j} \cdot \log (p_{j})$. Here, we assumed that we had *p* taxa in total, with *p_j_* being the relative abundance of taxon *j* in 1 sample. Then the α-diversities of the real and the simulated samples were compared through the Wilcoxon rank-sum test. We compared sample diversity using the β-diversity with the unweighted UniFrac metric [23]. The unweighted UniFrac distance between a pair of samples *m* and *k* is defined as *U_mk_* = unique/observed, where "unique" and "observed" represent the unique and the total branch length in sample *m* or sample *k*. The β-diversities were visualized by nMDS.

We used Spearman correlation coefficients and proportionality between taxa to measure the taxa-taxa relationships. We excluded taxa with an excessive number of zeros (>90%) across all real samples. Then the pairwise Spearman correlation coefficients or the “goodness-of-fit proportionality” statistic were calculated among all the remaining taxa.

### Online resource for MB-GAN

We used Keras [32] with the Tensorflow [33] back end to implement the MB-GAN model. We provide the source codes, example output datasets, and a Jupyter Notebook in GitHub as an online resource (https://github.com/zhanxw/MB-GAN). These include the trained models for the generator network, and they can facilitate reproducing the results reported in this article. They can also be customized to simulate new datasets for future microbiome studies.

## Availability of Source Code and Requirements

- Project name: MB-GAN
- Project home page: https://github.com/zhanxw/MB-GAN
- Operating systems: Linux
- Programming language: Python (version 3.6.8), R (version 3.6.0)
- Other requirements: Tensorflow (version 1.14.0), Keras (version 2.2.4)
- License: GNU General Public License v3.0
- RRID:SCR_019289
- biotoolsID identifier: biotools:mb-gan

## Data Availability

The Python code to implement MB-GAN, the R code for NorTA and metaSPARSim simulation, and the codes to reproduce all the figures and tables are openly available in the Github repository https://github.com/zhanxw/MB-GAN. Snapshots of our code and other supporting data are openly available in the *GigaScience* repository, GigaDB [34]. The original sequencing data from the fecal samples are available in the European Nucleotide Archive (ENA) database with the study number PRJEB1220.

## Additional Files

Supplementary Figure S1. Heatmaps of abundances of the 60 most abundant taxa extracted from the real data.

Supplementary Figure S2. Case group: Box plots of the abundances of taxa with less than 10% of zeros and 10%-20% of zeros in the real data of the case group.

Supplementary Figure S3. Control group: Box plots of the abundances of taxa with less than 10% of zeros and 10%-20% of zeros in the real data of the control group.

Supplementary Figure S4. Box plots of the abundances of taxa with less than 10% of zeros and 10%-20% of zeros in the real data.

Supplementary Figure S5. Box plots of Shannon index calculated from three datasets (Real, MB-GAN, Normal-To-Anything (NorTA), and metaSPARSim).

Supplementary Figure S6. β-diversity visualization using non-metric multidimensional scaling (nMDS) for the CRC and the control group.

Supplementary Figure S7. Correlogram of Spearman’s correlation coefficients calculated from the top 10% most abundant species in the case group.

Supplementary Figure S8. Correlogram of Spearman’s correlation coefficients calculated from the top 10% most abundant species in the control group.

Supplementary Figure S9. Scatter plot of the Spearman correlation coefficients between the real and the simulated data by MB-GAN, Normal-To-Anything (NorTA), and metaSPARSim.

Supplementary Figure S10. Case group: Comparison of correlograms and empirical distributions of the Spearman correlation coefficients calculated from the real data and the three types of simulated data.

Supplementary Figure S11. Control group: Comparison of correlograms and empirical distributions of the Spearman correlation coefficients calculated from the real data and the three types of simulated data .

Supplementary Figure S12. Comparison of correlograms of the Spearman correlation coefficients calculated from the real data and the three types of simulated data.

Supplementary Figure S13. Heatmaps showing the pattern of proportionality calculated from the top 10% most abundant taxa of real data, and proportionality of the simulated data by MB-GAN, Normal-To-Anything, and metaSPARSim.

Supplementary Figure S14. Scatter plot of the “goodness-of-fit proportionality" statistic between the real and the simulated data by MB-GAN, Normal-To-Anything (NorTA), and metaSPARSim.

Supplementary Figure S15. Box plot of the rank of p-values for the top 10 differentially abundant species selected based on the real data. Each box contains the ranked p-values from the Wilcoxon rank-sum test after adding in 400 MB-GAN samples for the corresponding species.

Supplementary Figure S16. Type-I error and power of MiRKAT based on different kernels calculated from the MB-GAN simulated data.

Supplementary Figure S17. The phylogenetic transformation function.

Supplementary Figure S18. The architecture for the generator and critic model.

Supplementary Table S1. Sample sparsity calculated from four datasets (Real, MB-GAN, Normal-To-Anything (NorTA), and metaSPARSim).

Supplementary Section 1: Evaluating MB-GAN on a Smaller Microbiome Dataset

Supplementary Section 2: Differential Abundance Analysis by MB-GAN

Supplementary Section 3: Using MB-GAN to Design the Simulation Study of Evaluating MiRKAT

## Abbreviations

EM: Earth Mover; ENA: European Nucleotide Archive; GAN: generative adversarial network; MB-GAN: microbiome generative adversarial network; MSE: mean square error; MWAS: metagenome-wide association studies; nMDS: non-metric multidimensional scaling; NorTA: Normal-to-Anything; WGAN: Wasserstein generative adversarial network; WGAN-GP: Wasserstein generative adversarial network with gradient penalty; ziNB: zero-inflated negative binomial.

## Competing Interests

The authors declare that they have no competing interests.

## Funding

This work was supported by the National Institutes of Health (5P30CA142543, 5R01GM126479, 5R01HG008983, 1R56HG011035).

## Authors' Contributions

R.R. and S.J. performed the experiment. L.X., G.X., Y.X., D.J.L., and Q.L. provided resources and helpful discussions. R.R., S.J., and X.Z. designed the experiment, performed data analysis, wrote the software, and wrote the manuscript.

## Supplementary Material

giab005_GIGA-D-20-00286_Original_Submission

giab005_GIGA-D-20-00286_Revision_1

giab005_GIGA-D-20-00286_Revision_2

giab005_Response_to_Reviewer_Comments_Original_Submission

giab005_Response_to_Reviewer_Comments_Revision_1

giab005_Reviewer_1_Report_Original_Submission Karel SedlÃ¡Å™ -- 10/7/2020 Reviewed

giab005_Reviewer_2_Report_Original_Submission Edoardo Pasolli -- 11/16/2020 Reviewed

giab005_Supplemental_File
