# Supplementary material for: MB-GAN: Microbiome Simulation via Generative Adversarial Network
Source: Gigascience. 2021 Feb 5;10(2):giab005. doi: 10.1093/gigascience/giab005 (PMC7931821; doi:10.1093/gigascience/giab005)

GigaScience

MB-GAN: Microbiome Simulation via Generative Adversarial Network

--Manuscript Draft--

**Manuscript Number:**	GIGA-D-20-00286R1
**Full Title:**	MB-GAN: Microbiome Simulation via Generative Adversarial Network
**Article Type:**	Research
**Funding Information:**	National Institutes of Health (5R01GM126479) — Dr Xiaowei Zhan<br>National Institutes of Health (5R01HG008983) — Dr Xiaowei Zhan
**Abstract:**	Background: Trillions of microbes inhabit the human body and have a profound effect on human health. The recent development of metagenome-wide association studies (MWAS) and other quantitative analysis methods accelerate the discovery of the associations between human microbiome and diseases. To assess the strengths and limitations of these analytical tools, simulating realistic microbiome datasets is critically important. However, simulating the real microbiome data is challenging since its correlation structure is difficult to be modeled by explicit statistical models.<br><br>Results: To address the challenge of simulating realistic microbiome data, we designed a novel simulation framework termed MB-GAN, by using a generative adversarial network (GAN) and utilizing methodology advancements from the deep learning community. MB-GAN can automatically learn from a given microbial abundances and compute simulated abundances that are indistinguishable from it. In practice, MB-GAN showed the following advantages. First, MB-GAN avoids explicit statistical modeling assumptions, and it only requires real datasets as inputs. Second, unlike the traditional GANs, MB-GAN is easily applicable and can converge efficiently.<br><br>Conclusion: By applying MB-GAN to a case-control gut microbiome study of 396 samples, we demonstrated that the simulated data and the original data had similar first-order and second-order properties, including sparsity, diversities, and taxa-taxa correlations. These advantages are suitable for further microbiome methodology development where high fidelity microbiome data are needed.
**Corresponding Author:**	Xiaowei Zhan<br>University of Texas Southwestern Medical School<br>Dallas, TX UNITED STATES
**Corresponding Author Secondary Information:**	
**Corresponding Author's Institution:**	University of Texas Southwestern Medical School
**Corresponding Author's Secondary Institution:**	
**First Author:**	Ruichen Rong
**First Author Secondary Information:**	
**Order of Authors:**	Ruichen Rong<br>Shuang Jiang<br>Lin Xu<br>Guanghua Xiao<br>Yang Xie<br>Dajiang Liu<br>Qiwei Li<br>Xiaowei Zhan

Powered by Editorial Manager® and ProduXion Manager® from Aries Systems Corporation

Order of Authors Secondary Information:	
Response to Reviewers:	Dear editor and reviewers,<br><br>We have uploaded a PDF file as respond. It has better formats to ease your reading. Thank you.
Additional Information:	
Question	Response
Are you submitting this manuscript to a special series or article collection?	No
**Experimental design and statistics**<br><br>Full details of the experimental design and statistical methods used should be given in the Methods section, as detailed in our Minimum Standards Reporting Checklist. Information essential to interpreting the data presented should be made available in the figure legends.<br><br>Have you included all the information requested in your manuscript?	Yes
**Resources**<br><br>A description of all resources used, including antibodies, cell lines, animals and software tools, with enough information to allow them to be uniquely identified, should be included in the Methods section. Authors are strongly encouraged to cite Research Resource Identifiers (RRIDs) for antibodies, model organisms and tools, where possible.<br><br>Have you included the information requested as detailed in our Minimum Standards Reporting Checklist?	Yes
**Availability of data and materials**<br><br>All datasets and code on which the conclusions of the paper rely must be either included in your submission or deposited in publicly available repositories (where available and ethically	Yes

appropriate), referencing such data using a unique identifier in the references and in the “Availability of Data and Materials” section of your manuscript.<br><br>Have you have met the above requirement as detailed in our Minimum Standards Reporting Checklist?	

Manuscript

Click here to access/download;Manuscript;main_mbgan.pdf

GigaScience, 2017, 1–11

doi: xx.xxxx/xxxx
Manuscript in Preparation
Research

RESEARCH

MB-GAN: Microbiome Simulation via Generative Adversarial Network

Ruichen Rong[1,†], Shuang Jiang[1,2,†], Lin Xu[1], Guanghua Xiao[1], Yang Xie[1], Dajiang J. Liu[3], Qiwei Li[4] and Xiaowei Zhan[1,*]

[1]University of Texas Southwestern Medical Center, Dallas, Texas, 75390, USA and [2]Southern Methodist University, Dallas, TX 75275, USA and [3]Pennsylvania State University, Hershey, Pennsylvania, 17033, USA and [4]The University of Texas at Dallas, Dallas, Texas, 75080, USA

[*]Xiaowei.Zhan@utsouthwestern.edu
[†]Contributed equally.

Abstract

Background: Trillions of microbes inhabit the human body and have a profound effect on human health. The recent development of metagenome-wide association studies (MWAS) and other quantitative analysis methods accelerate the discovery of the associations between human microbiome and diseases. To assess the strengths and limitations of these analytical tools, simulating realistic microbiome datasets is critically important. However, simulating the real microbiome data is challenging since its correlation structure is difficult to be modeled by explicit statistical models. **Results:** To address the challenge of simulating realistic microbiome data, we designed a novel simulation framework termed MB-GAN, by using a generative adversarial network (GAN) and utilizing methodology advancements from the deep learning community. MB-GAN can automatically learn from a given microbial abundances and compute simulated abundances that are indistinguishable from it. In practice, MB-GAN showed the following advantages. First, MB-GAN avoids explicit statistical modeling assumptions, and it only requires real datasets as inputs. Second, unlike the traditional GANs, MB-GAN is easily applicable and can converge efficiently. **Conclusion:** By applying MB-GAN to a case-control gut microbiome study of 396 samples, we demonstrated that the simulated data and the original data had similar first-order and second-order properties, including sparsity, diversities, and taxa-taxa correlations. These advantages are suitable for further microbiome methodology development where high fidelity microbiome data are needed.

Key words: Microbiome simulation; Generative adversarial network; Deep learning.

Background

The microbiome is a collection of trillions of microorganisms living within humans. Previous studies have revealed that the microbiome has a profound impact on human disease, including inflammatory bowel disease, colorectal cancer, type-2 diabetes, and psychiatric disorders [1, 2, 3, 4]. A powerful and increasingly popular method to study the microbiome and disease is metagenome-wide association studies (MWAS). These studies utilize the taxonomic abundance data of thousands of bacteria generated from sequencing instruments, and calculate the associa-

Compiled on: November 23, 2020.
Draft manuscript prepared by the author.

tion strengths between the bacterial abundances and the phenotypes. As researchers have expanded their interests into studying microbial associations in human physiology, MWAS has taken on an increasingly critical role.

A successful MWAS relies on valid statistical models [5], and the evaluation of MWAS models relies on simulations. As for methods development, designing benchmark settings that fully capture the characteristics of the microbiome data could reasonably reflect the qualities across various models. For example, Jiang et al. [6] demonstrated that `edgeR`, a statistical model proposed for analyzing the RNA-seq data [7], showed inferior performance on data with a much higher sparsity such as microbiome data. Furthermore, a reasonable summary of the model performance based on simulation guides real-world applications. In practice, the user can specify realistic scenarios in simulation, and select the best model with the highest empirical statistical power.

Though simulation plays an vital role in MWAS, it is not trivial to simulate microbiome abundances with high fidelity to the real data. Microbiome abundances are sparse, over-dispersed (large variances compared to means), and have an intrinsic phylogenetic relationship [8]. In addition, as the microbiome consists of interactive communities, the microbiota form complex taxa-taxa relationships with a noneligible second-order covariation [9, 10]. However, a simulation method that can capture the first order (e.g., sample-level) characteristics while maintaining the second order (e.g., taxa-taxa level) relationships is lacking in the current literature. For example, explicit statistical distributional assumptions, such as the zero-inflated logistic normal distribution, were introduced to simulate individual bacterial taxon [11]. Although the simulated data have the desired sample-level and taxa-level properties (e.g., sparsity and overdispersion), they ignore the taxa-taxa relationships. Other methods, such as Normal-To-Anything (NorTA) [12], have attempted to model the taxa-taxa relationships, but their performance at the sample-level is not satisfactory, as we will demonstrate later in this manuscript.

Given the above challenges in explicitly modeling the microbiome abundances, we developed a novel deep learning-based approach to implicitly compute simulated microbial abundances with desired sample-level and taxa-taxa interactive characteristics. We refer to this simulation framework as MB-GAN (Microbiome Generative Adversarial Network), as it is adapted from a Generative Adversarial Network (GAN) framework [13] and is customized for simulating microbiome datasets. GAN has been a feature of deep learning studies since its foundational work proposed in 2014 [13]. It has a generator network and a discriminator network. The generator network takes random noise and outputs simulated data. The discriminator network takes both the simulated and the real datasets, and classifies the inputs as real or fake. In the network training stage, the generator focuses on increasing the similarity between the simulated data and the real data, meanwhile, the discriminator works on better distinguishing between the simulated and the real data. When the training stage finishes, the generator will be able to simulate data that are hard for the discriminator to distinguish from the real data. Due to the excellent performance of GANs compared with conventional approaches (e.g., variational auto-encoder [14]), the GAN-based models have wide applications, such as image synthetization (e.g., human facial images [15]), text generation (e.g., visual paragraph generation [16]) and music synthetization (e.g., music composition [17]). Recently, GAN models have also been adapted for biomedical research. For example, GAN models have been applied in generating sequence data (e.g., t-cell receptor sequences [18]) and enhancing medical imaging [19]. Given the lacking of performant simulation models for microbiome datasets and the impressive potential of the GAN-based simulation models, we are motivated to incorporate the GAN model in order to simulate microbiome abundances.

In this manuscript, our contribution is a novel microbiome simulation model, MB-GAN, and showed it can simulate high fidelity microbiome abundances. Specifically, we modified the discriminator network to incorporate the microbiome diversity-based measurements. Compared to the original GAN framework, our algorithm converges fast and robustly. It is thus easily applicable to simulate new datasets based on a set of input microbiome abundances without explicit modeling. In a real data study, we demonstrated that the simulated microbiome abundances have similar data characteristics, including both of the first order (sample-level properties such as sparsity and diversity) and the second order properties (taxa-taxa correlations). Thus the simulated data can be utilized in further methodology development and evaluations.

Data description

We benchmarked the MB-GAN model using a real sequencing dataset from a human gut microbiome study published by Nielsen et al. [20]. The dataset contains 396 sequenced shotgun metagenomic samples with 148 inflammatory bowel disease (IBD) patients and 248 healthy controls. The original sequencing data from the fecal samples are available in the European Nucleotide Archive (ENA) database with the study number PRJEB1220.We used `curatedMetagenomicData` [21] to obtain the taxonomic abundance table of all samples with 1, 939 detected taxa at different taxonomic levels. We further separated the samples into case (IBD patients) and control (healthy controls) groups to implement MB-GAN separately (see more details in Method section). We also used the phylogenetic tree accompanied to the original sequencing data provided by `curatedMetagenomicData`. In addition, results from MB-GAN on a microbiome study with smaller sample sizes (111 in total) are available in section "Evaluating MB-GAN on a Smaller Microbiome Dataset" in the supplement.

Analyses

To simulate microbiome abundances using MB-GAN, we used the 148 cases (or 248 controls) as real data input to train the MB-GAN. We set 100, 000 iterations with a batch size of 32 (see more details in Method section). We simulated 1, 000 MB-GAN samples for each group separately, and both the generators and critics reached convergence in 10 minutes after 20, 000 iterations. For a brief illustration of how similar the MB-GAN samples were to the real ones, we picked the 60 most abundant taxa from the 148

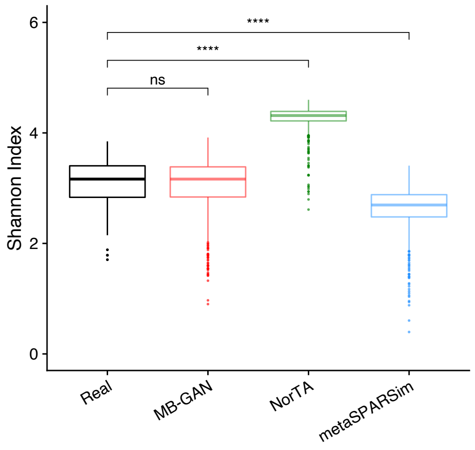

(a) Shannon index of the case group

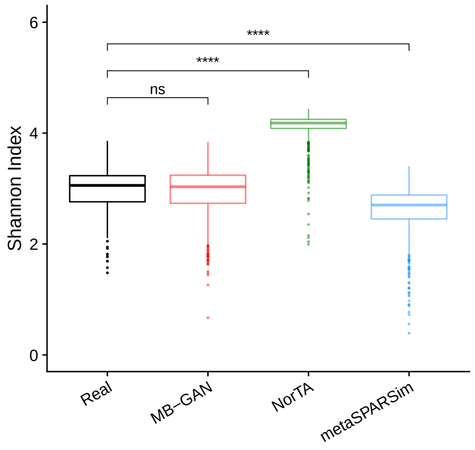

(b) Shannon index of the control group

Figure 1. Box plots of Shannon index calculated from three datasets (Real, MB-GAN, Normal-To-Anything (NorTA), and metaSPARSim) under (a) case or (b) control group.

case samples, and compared their abundances between the real and simulated data. As a comparison, we also considered two additional simulation methods: Normal-To-Anything (NorTA) and metaSPARSim [12, 22]. NorTA was designed to generate multivariate random variables with a pre-specified correlation structure, and metaSPARSim was a model-based approach to simulate 16S rRNA sequencing count data. More details were introduced in the Method section. Briefly speaking, we implemented NorTA to the same real dataset, where we generated 1,000 samples based on the 148 case and 248 control samples, respectively. Similarly, we utilized metaSPARSim to simulate microbiome abundances of the same sample sizes. By comparing the performances of MB-GAN and other methods, we demonstrated the high-fidelity of MB-GAN samples to the real data (Figure S1(a), (b)), as there are similar patterns of abundances shared between the real datasets and MB-GAN simulated datasets. We further compared the simulated and observed abundances to examine the fidelity of the MB-GAN samples in different taxa abundance strata. In the case group, we considered (1) the taxa having less than 10% zeros across all the samples, and (2) the taxa having 10% $\sim$ 20% zeros across all the samples. In each scenario, we compared the observed and simulated abundances (by MB-GAN and other methods) using the Wilcoxon rank-sum test. The MB-GAN samples gave p-values > 0.05 in both scenarios, whereas the NorTA and metaSPARSim samples showed significantly different abundances (both p-values < 0.0001) when compared with the observed data for those abundant taxa (Figure S2). We performed the same analysis on the control group. The p-values are 0.89 for scenario (1) and 0.031 for (2) when testing the MB-GAN simulated abundances against the observed ones. Again, the p-values by the NorTA and metaSPARSim results were less than 0.0001 (Figure S3). This showed that the MB-GAN was able to simulate well the highly abundant taxa in the real data. In contrast, the NorTA simulated abundances are smaller in the magnitude compared to both the observed and the MB-GAN abundances, and the metaSPARSim did not perform well either. We concluded that neither NorTA nor metaSPAR shared a good pattern with the observed abundances. We performed the same analysis on the smaller sample size dataset and reached the same conclusions. As shown in Figure S4, the Wilcoxon rank-sum test for the MB-GAN samples yielded p-values > 0.05 for the case and control groups, while the p-values by the NorTA and metaSPARSim results were less than 0.0001.

Evaluation on sample-level properties

First, we evaluated sample sparsity, which is the proportion of zeros in a sample. For the real data, the observed sparsity ranges from 0.71 to 0.90, with median values being 0.80 and 0.83 for the case and the control group, respectively. As for the two types of simulated data, the lower bound of sample sparsity by MB-GAN matched well with the real data for both groups, but in general the MB-GAN simulated data showed a slightly higher sparsity with a median of 0.83 and 0.85 for the case and the control group. NorTA simulated data, on the other hand, tended to underestimate the sparsity in both case and control groups. The median values of the sample sparsity were all below 0.80 for the two groups, and the maximum sparsity was less than 0.85. The sparsity given by metaSPARSim was also lower than the actual values, in general. The overall sparsity by metaSPARSim ranged from 0.71 to 0.84, with the median being 0.77 and 0.80 for the case and the control group, respectively. The maximum sparsity was only 0.84, which was smaller than the observed maximum (0.90) from the real data. Thus, MB-GAN simulated data could better capture the sparsity observed across all the samples of the real data. This conclusion also held for the smaller sample size case, as shown in Table S1.

Next, we evaluated the α-diversities of the simulated data from MB-GAN, NorTA, and metaSPARSim. We compared the Shannon indices calculated from the simulated and the real samples. The Shannon index is a metric that weights the relative abundance of species by their relative evenness in a sample (see more details in Method section). As an α-diversity index, it provides more information than simply species richness (i.e., the number of

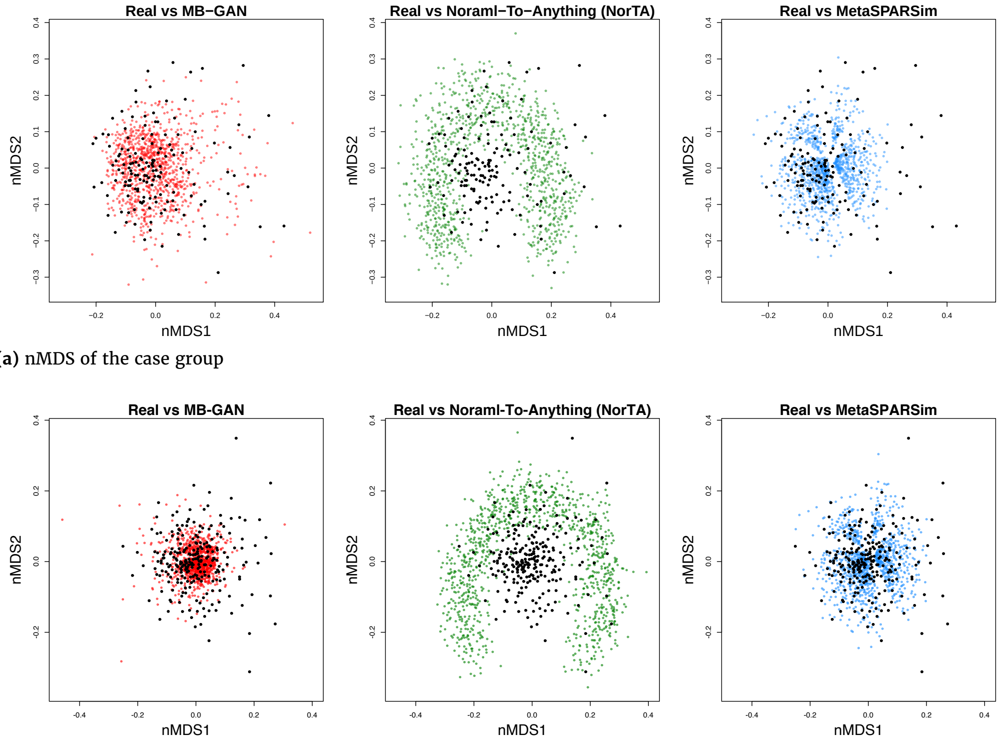

(a) nMDS of the case group

(b) nMDS of the control group

Figure 2. β-diversity visualization using non-metric multidimensional scaling (nMDS) for (a) the case and (b) the control groups. For samples from the real data, MB-GAN, Normal-To-Anything (NorTA), and metaSPARSim simulated data, the two-dimensional nMDS values were calculated using the unweighted UniFrac metric.

species in the sample) since it considers the relative abundances of different species. Therefore, a better matching between the Shannon indices calculated from the simulated data and the real data suggested that the simulator could better characterize the biodiversity of the real data. As shown by the box plots in Figure 1(a) and (b), the Shannon indices calculated from MB-GAN simulation data matched consistently with the real data in both the case and the control groups. The p-value given by the Wilcoxon rank-sum test was 0.83 for the case group and 0.57 for the control group. As for the two alternative methods, the Shannon indices by NorTA were larger than the real ones, and the variation among the results was not well-characterized. This suggests that the samples simulated by NorTA were less diverse compared to the real data. In contrast, the Shannon indices by metaSPARSim were smaller, suggesting a higher diversity in the metaSPARSim samples than the real data. Again, the Wilcoxon rank-sum test yielded p-values less than 0.0001 for both groups when comparing the real data against NorTA or metaSPARSim simulated data. The box plots in Figure S5 for the smaller sample size case also demonstrated the same conclusion. In all, our results suggested that the MB-GAN simulated data better resembled the real microbiome abundances with respect to α-diversity.

Finally, we calculated the β-diversity of the simulated data from MB-GAN, NorTA and metaSPARSim, and compared the results with the β-diversity of the real data. β-diversity measures how different samples are from each other (see more details in Method section), and a commonly used way to visualize β-diversity is by non-metric multidimensional scaling (nMDS) analysis. Here, the samples were compared based on their species level abundances. We incorporated the species' phylogenetic information by using UniFrac distance [23] matrix to generate the nMDS plots. Figure 2(a) and (b) visualized the results from the case and the control group, respectively. In each subfigure, the black dots represented the results from the real data, and different colored dots represented the results from three simulators. For both groups, the clear overlap between the real data and the MB-GAN simulated data demonstrated the similarity between those samples. The NorTA simulated data, however, showed a unique circular pattern that was different from either the real data or the MB-GAN simulated data. The results given by metaSPARSim were less spread than the real data. Meanwhile, the points formed smaller groups separated by the gaps shown in the third column of Figure 2. Note that these gaps were not observed for the real data. Interestingly, the aforementioned patterns were also observed for the smaller sample size case, as shown in Figure S6. Compared to the two alternative methods, data generated by MB-GAN demonstrated a more reasonable representation of the real data in terms to the UniFrac β-diversity.

Evaluation on taxa-taxa relationships

In addition to comparing the sample-level similarities, we illustrated that MB-GAN had the advantage of preserving

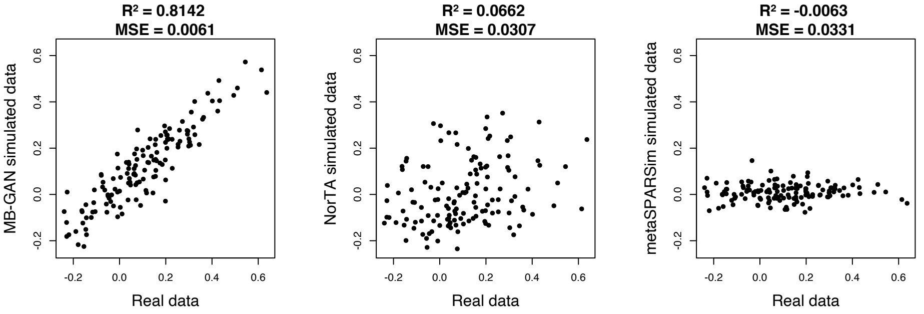

(a) Case group: Spearman correlation coefficients between the real and the three types of simulated data using the top 10% most abundant species from the real data (from case group)

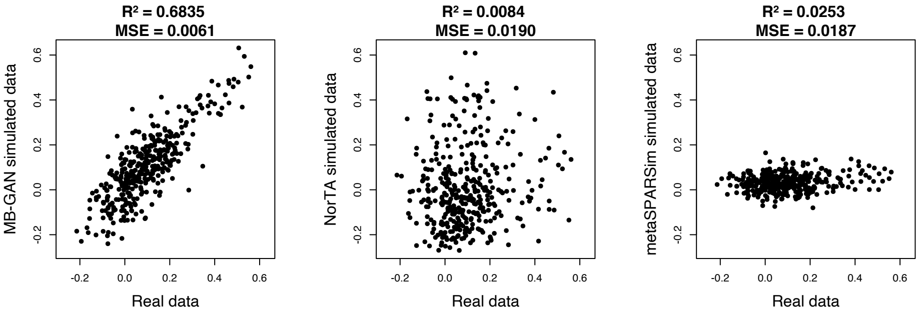

(b) Control group: Spearman correlation coefficients between the real and the three types of simulated data using the top 10% most abundant species from the real data (from control group)

Figure 3. Scatter plot of the Spearman correlation coefficients between the real and the simulated data by MB-GAN, Normal-To-Anything (NorTA), and metaSPARSim. Results are calculated based on the top 10% most abundant species from the real data of the (a) case and the (b) control group, respectively.

the second order characteristics in the real data. We measured the taxa-taxa relationships using (1) Spearman's correlation coefficients, and (2) proportionality between taxa pairs. We visualized the correlation matrices and the empirical distributions of the correlation coefficients to compare the real data and the three types of simulated data (MB-GAN, NorTA, and metaSPARSim). Here, the comparison considered the top 10% most abundant species in the real data, since these taxa contained more information in capturing the taxa-taxa interactions. The names of the remaining species can be found in Figure S7 and S8 for the case and the control group, respectively.

The scatter plots in Figure 3(a) and (b) visualized the Spearman's correlations calculated from the real data against the simulated data. The R^2 and mean square error (MSE) were included in the plot. In general, there was a relatively strong linear trend in the first subfigure of Figure 3(a), (b), suggesting that the correlation structure from MB-GAN samples resembled that of the real data for both groups. Whereas for the other two simulators, the Spearman's correlations seemed to be quite different from the real data, as shown in the second and third subfigure in Figure 3(a), (b). The results given by metaSPARSim suggested that the simulated taxa had weak association measured by the Spearman's correlation coefficient. As for the smaller sample size case, Figure S9 also demonstrates that MB-GAN samples better preserved the correlation structure in the real data. Figure S10(a) and S11(a) compare the patterns in correlograms of Spearman's correlation matrices, calculated from the real and simulated data. A blue ellipse represents a negative correlation, while a red one suggests a positive correlation. The darker the color, or the shorter the ellipse's minor axis, the stronger the correlation between the corresponding taxa pair. MB-GAN was able to capture the overall pattern of the correlogram from the real data, and it preserved the relatively strong associations for both the case and the control groups. Same conclusion held for the smaller sample size case, as shown in Figure S12. However, all the three simulators tended to show weaker associations compared to the real data, especially for metaSPARSim. Further, we observed a clear disparity between the correlograms by NorTA and the real data. Figure S10 (b) and S11 (b) overlay the empirical distributions of the Spearman's correlation coefficients from the real data and a type of simulated data. For both the case and the control group, the empirical distributions given by MB-GAN better matched the true coefficients' distributions. In all, Figure 3, Figure S10, and Figure S11 illustrated the overall better performance of MB-GAN over NorTA and metaSPARSim with respected to capturing the

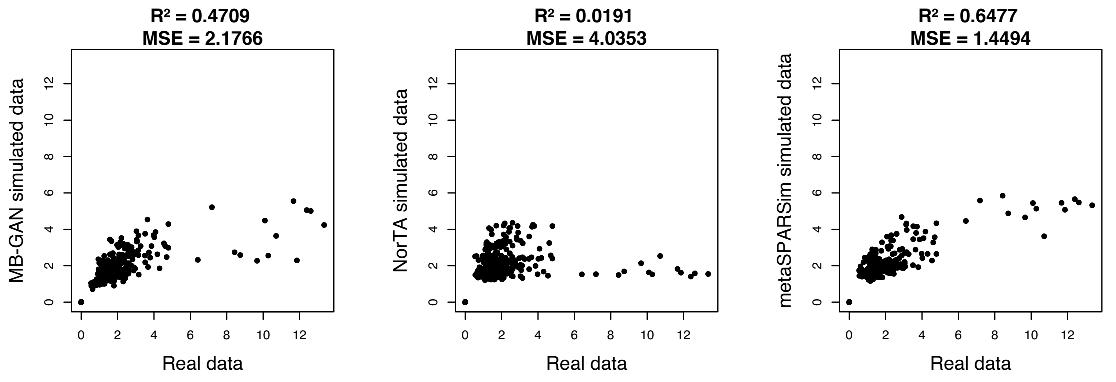

(a) Case group: Proportionality between the real and three types of simulated data using the top 10% most abundant species from the real data

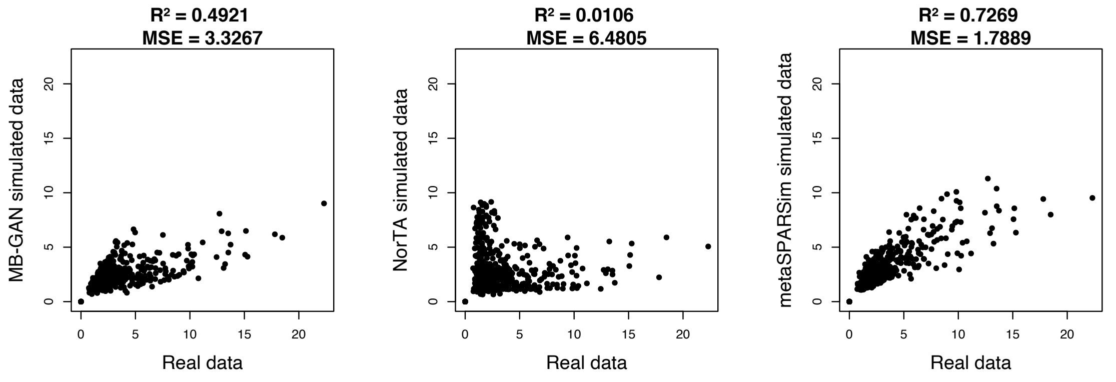

(b) Control group: Proportionality between the real and three types of simulated data using the top 10% most abundant species from the real data

Figure 4. Scatter plot of the "goodness-of-fit proportionality" statistic between the real and the simulated data by MB-GAN, Normal-To-Anything (NorTA), and metaSPARSim. Results are calculated based on the top 10% most abundant species from the real data of the (a) case and the (b) control group, respectively.

taxa-taxa relationships measured by Spearman's correlation.

In addition to the Spearman's correction, we further compared the taxa-taxa relationships using the idea of "proportionality" proposed by Lovell et al. [24]. Proportionality was designed to analyze relative data. It had the advantage of preserving the associations between a pair of abundances for taxon m and k across all samples, (x_m, x_k), in their absolute scale. We calculated the "goodness-of-fit to proportionality" statistic ϕ as $\phi(\log x_m, \log x_k) = \mathrm{var}(\log(x_m/m_k))/\mathrm{var}(\log x_m)$, as suggested by Lovell et al. [24]. Further, it was recommended to apply the *centered logratio* (clr) transformation on sample-level to ensure the results between different variable pairs are comparable. Specifically, $\mathrm{clr}(x_m) = \log \frac{x_m}{g(x)}$, with $g(x)$ being the geometric mean of the sample x.

We presented the results using scatter plots to examine Lovell's proportionalities for the case group and the control group for all simulation methods (Figure 4). We also calculated the correlation of coefficients (R^2) and mean square errors (MSE) based on all points in each sub figure. We found that higher R^2 values and less MSEs for the MB-GAN and metaSPARSim compared with the NorTA. Additionally, based on the heatmaps in Figure S13, we observed that both MB-GAN and metaSPARSim shared a lot of similarities with the heatmaps of real data for both the case and the control groups. These observations also held when we used a smaller sample size (Figure S14). In general, MB-GAN and metaSPARSim performed reasonably well in preserving the proportionality of real data. Notably, as shown in the previous section, MB-GAN could outperform metaSPARSim by preserving sample-level properties of the real data.

Discussion

While MWAS greatly facilitate the investigation of association between the human microbiome and diseases, the evaluation of existing MWAS models requires realistic simulation studies. However, it is challenging to specify explicit statistical distributions in simulation to fully mimic the complex patterns observed in the real microbiome data. Specifically, few simulation frameworks achieve good performance in both modeling the sample-level characteristics (e.g., sparsity, α-diversity, and β-diversity, etc.) and maintaining realistic levels of taxa-taxa associations. To address these challenges, we have developed a novel simulation framework, MB-GAN. It is designed based on the latest research into GANs and adapted the phylogenetic transformation and ecologically meaningful discriminator loss for improved convergence. The simulator is trained by real data and does not need explicit statistical models. It can simulate microbiome relative abundances that are not easily distinguishable from real data in terms of sample-level characteristics and taxa-taxa relationships. For example, MB-GAN can mimic original data and provide similar simulated relative abundances in terms of diversity, sparsity, and feature correlations. If a small dataset is not sufficient to train a converged MB-GAN model, we recommend checking the data quality and network structure first and then exploring the adaptive learning strategies to gradually adding new samples into the model till newly generated data could represent the original training data.

Recently, GAN has been widely adapted to generate simulated data that are hard to distinguish from real data. Researchers have successfully applied GANs to different fields, including text generation, music synthetization, and image synthetization. GANs have also been adapted for biomedical researches such as generating sequence data. Our proposed method, MB-GAN, is to our knowledge the first simulation framework that adapts GANs to generate microbiome abundances. Unlike the traditional GANs, our algorithm is easily applicable and can converge efficiently.

However, MB-GAN should not be used as a tool to enlarge existing sample sizes in MWAS. For example, detecting differentially abundant taxa based on MB-GAN enlarged samples may not yield valid biological conclusions. We performed a simple differential abundance analysis in the supplement (Section "Differential Abundance Analysis by MB-GAN"). The results shown in Figure S15 suggested that it may be invalid to draw biologically meaningful conclusion based on the mixed data consisting of both the MB-GAN simulated samples and the real samples. Alternatively, we recommend using MB-GAN to design the simulation study and evaluate MWAS models. These models can be critically important to understand the relationship between microbiome and health in the future.

Potential implications

MB-GAN can benefit future quantitative methodology development for microbiome research. For example, the MB-GAN simulated data can be used to evaluate statistical models designed for MWAS studies: researchers can use MB-GAN to simulate microbiome abundances for a certain sample size and impose the statistical effect sizes on a subset of taxa for different phenotype groups [25], perform analysis using these MWAS models, and classify the detected differentially abundant taxa into true positives and the false positives. In this way, researchers can examine the Type-I error (false positive) and power of these MWAS models. A concrete example of using MB-GAN sample to evaluate MiRKAT Zhao et al. [25], a widely-used statistical model proposed for MWAS, is available in section "Using MB-GAN to Design a Simulation Study to Evaluate MiRKAT" in the supplement. Figure S16 summarized the Type-I error and statistical power of MiRKAT based on the simulation designed by using MB-GAN sample. In addition, the investigations of microbiome networks can also utilize MB-GAN since MB-GAN can effectively preserve the taxa-taxa interactions compared to other existing approaches. Statistical models that focus on detecting the correlation-based microbiome co-occurrence pattern can use the MB-GAN samples for model evaluation. In conclusion, MB-GAN enables the evaluation of various types of microbiome studies by providing simulated data with high fidelity to the real data.

Methods

Construction of MB-GAN

We created MB-GAN using a generator network and a discriminator network, which resembled the classic GAN network. The goal of the generator network is to take random noise, conduct a series of non-linear transformation, and compute simulated microbiome relative abundances. The goal of the discriminator is to distinguish whether the data are from generator or from the real datasets. While the MB-GAN architecture was similar to the GAN framework, we designed two specific layer to allow it better usable for microbiome data. First, we added a phylogenetic transformation layer. As many microbial taxa have low abundances, we can transform it to larger values in order to achieve better model convergence. Second, we used Earth Mover's (EM) distance to compute the loss function for the discriminator network. This can quantitatively measure the similarity between any two microbiome samples given their phylogenetic information. The computed diversity value was used as the loss value in the discriminator network.

The usability of MB-GAN network was founded by the Wasserstein GAN with gradient penalty (WGAN-GP) framework [26] which exhibited several improvements over the classic GAN framework [13]. Briefly speaking, the classic GAN has two major drawbacks: 1) the generator stops updating when discriminator is overpowered (e.g., generator's gradients vanish or explode); 2) generated samples lack enough variability (model collapse [27]). To overcome these problems and fully unleash the power of GAN, WGAN was proposed [28]. It invented a scalar similarity score (e.g., the Earth Mover's (EM) distance) as a critic to measured the quality of the simulated data, and replaced the original binary discriminator that distinguished the data are real or fake by probabilities. In both theory and practice, WGAN provided a smoother gradient everywhere and thus the generator continued to learn new knowledge even when the critic already performed well.

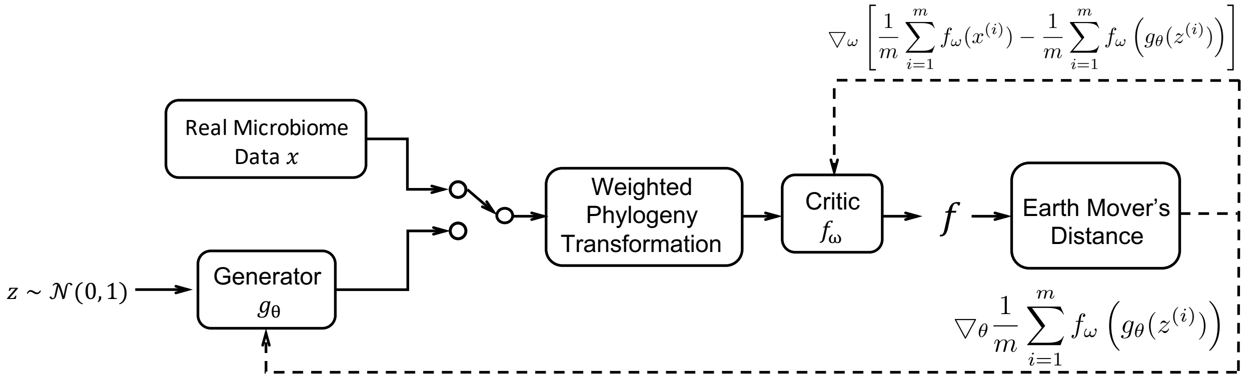

Figure 5. The MB-GAN network architecture. For the solid lines: the Gaussian noise z is passed to the generator model g_θ; both real microbiome data x and simulated microbiome data $g_\theta(z)$ undergo weighted phylogeny transformation and are passed to the critic model f_ω; the function f calculates the Earth Mover's distance. For the dashed lines: the differentiated scores are used to update generator weights θ and critic weights ω at the i-th iteration.

Therefore, we utilized a scalar loss value computed by the EM distance in the MB-GAN discriminator network. The EM distance was introduced in the WGAN [28], so that the MB-GAN shared the same benefits as those from the WGAN. To further improve the model convergence and simulation quality, we incorporated the training strategy proposed in the WGAN-GP [26], which reformed the gradient clipping into a gradient penalty to solve a practical issue lead by WGAN's Lipschitz constraint. In our observation, training MB-GAN using this strategy showed no sign of any gradient problems or model collapse and thus was an optimized framework for training a well-performed GAN generator for simulation.

The detailed architecture of our proposed MB-GAN network structure is illustrated in Figure 5. In the training phase, the MB-GAN network requires a real microbiome dataset x, which contains the relative abundances of taxa observed across different samples. For each sample, the relative abundance of a taxon is defined as its percent composition relative to the total abundances observed in that sample. The generator takes random noise inputs (e.g., $z \sim \mathcal{N}(0,1)$ as Gaussian noise) and outputs simulated microbiome datasets ($g_\theta(z)$). Both real microbiome samples and simulated samples are combined in one batch. Then all data would undergo the weighted phylogeny transformation, and be sent to a critic to calculate the EM distance between the real and simulated samples. The generator and critic are differentiated against the distance alternatively to update the model weights.

To handle microbiome data and incorporate its underlying tree structure, we added a transformation layer. It integrates additional taxonomic information into the critic to biologically measure the dissimilarity between the real sample and the generated sample. First, the transformation layer expands the species-level table based on the hierarchical tree structure to make a full abundance matrix (e.g., aggregate all taxa abundances from phylum to species). When feeding the transformed matrix into the WGAN-GP framework, the critic calculates an EM distance. Notably, the EM distance here is often termed as Wasserstein-1 distance or Kantorovich-Rubinstein metric in the machine learning community [28], and it is not to be confused with the phylogenetic Kantorovich-Rubinstein metric [29]. Second, we can integrate algorithmic transformations into the transformation layer. For example, we can apply logarithmic transformation as a normalization step to our MB-GAN framework. In this manuscript, we transformed each observed relative abundance x into $\log \frac{1+1000x}{1+x}$. In practice, we observed that this transformation provided robust and satisfactory results regarding the first and second order properties, even without the help of tree branch length. Figure S17 in the supplement shows how the transformation amplifies the observed small abundances.

Implementation

Data processing

As illustrated in Figure 5 and Supplementary Figure S18, the input of the generator model is a Gaussian random noise vector, and the inputs of the critic model are the real data and the simulated data. Next, both the real and the simulated data go through the weighted phylogeny transformation, which utilizes a rooted taxonomic tree (or phylogenetic tree) to expand the species- (or OTU-) level abundance matrices. The outputs include the abundance of all internal nodes for each input sample. Last, the critic uses the outputs to calculate a Earth Mover's distance.

MB-GAN training

We incorporated the training method in the WGAN-GP with our own customization. For the critic, we use Wasserstein loss to measure the EM distance between the real data and the simulated data. Meanwhile, we employed the GP loss to achieve Lipschitz constraint required by the WGAN-GP. We weighted the Wasserstein loss and GP loss by 1:10. For the generator, we used Wasserstein loss to measure the minimum cost to change the simulated data into real data. In the MB-GAN training phase, we used RMSprop optimizer with learning rate 5×10^{-5} to update the model weights. The generator and critic are differentiated alternatively, with 5 steps of critic followed by 1 step of generator in each iteration.

We assessed the convergence of the MB-GAN using two statistics. The first statistic combined the Wasserstein loss of generator and the Wasserstein loss of critic. Indeed,

these losses can also be used for model selection. The second statistic was the mean of the mean square error (MSE) of the change of model parameters across iterations. The training of critic and generator was considered to reach convergence when the difference of the mean MSE was less than 1×10^{-8} in a 1000-iteration window. In practice, it is not necessary to stop the model training when the mean MSE is small. As the MB-GAN model shows no sign of collapse during the training, it is recommended to train the model as long as possible even when the model reaches convergence, and then the best model can be selected by comparing model performance.

Comparison and evaluation of the MB-GAN

Comparison with Normal-To-Anything

We compared the MB-GAN to Normal-To-Anything (NorTA), an alternative simulation methods for microbiome data. NorTA was designed to generate multivariate random variables with a pre-specified correlation structure [12]. In general, NorTA transforms a multivariate Gaussian random variable with a given correlation structure to an arbitrary discrete or continues random variable, where the transformation is determined by the marginal distribution of the target random variable. In recent microbiome network studies, the NorTA has been used to generate high-dimensional sparse count data with the underlying correlation structure defined by a target correlation matrix [30, 31]. Choosing the zero-inflated negative binomial (ziNB) as the target marginal distribution in the transformation step was shown to well characterize the overdispersion and zero-inflation observed in real microbiome data [30]. Briefly, the NorTA method includes the following steps: 1) remove the taxa with zeros across all real data samples; 2) generate an $n \times p$ multivariate normal random variable with zero mean and $p \times p$ correlation matrix calculated from the real data, where n and p are the sample size and taxa number, respectively; 3) for each taxon j, apply a standard normal cumulative distribution function transformation to get a uniform random variable; 4) apply the quantile function of a zero-inflated negative binomial (ziNB) distribution to each uniform random variable to generate the count vector for each taxon j, with the parameters of the ziNB distribution estimated from the observed count data of taxon j; 5) compositionalize the resulting $n \times p$ count matrix by dividing each sample (row) by the total counts in that sample. The above steps are implemented in the function `synth_comm_from_counts` in the R package `SPIEC-EASI` [30].

Comparison with metaSPARSim

metaSPARSim is a 16S rDNA-seq data simulator [22]. It simulates count matrices resembling real sequencing count data using a model-based approach. metaSPARSim consists of two steps. The first step models the variation of species abundances between biological replicates through a Gamma distribution. The second stop employs a multivariate hypergeometric model to capture the technical variability in the sequencing process. Patuzzi et al. [22] suggested that metaSPARSim was able to generate synthetic data resembling real 16S rDNA-seq data with respect to the compositionality and sparsity. The count matrices given by metaSPARSim could potentially serve for assessing the analytical tools for count data normalization and differential abundance analysis. In this manuscript, we used the functions `estimate_parameter_from_data` and `metaSPARSim` in the R package published at http://sysbiobig.dei.unipd.it/?q=metaSPARSim (version 1.1.1) to implement metaSPARSim. In total, 1,000 samples were generated for the case and the control group, respectively.

Evaluation of model performance

To evaluate the quality of the simulated datasets, we compared sample-level statistics and taxa-taxa correlations calculated from the real data and the two types of simulated data.

We used sparsity and diversity to measure the sample-level characteristics. The sparsity was defined as the proportion of zeros in a sample. In the MB-GAN outputs, abundances less than 1×10^{-4} were truncated to zero. The α-diversity for each sample was defined as the Shannon index: $-\sum_{j=1}^{p} p_j \cdot \log(p_j)$. Here, we assumed that we had p taxa in total, with p_j being the relative abundance of taxon j in one sample. Then the α-diversities of the real and the simulated samples were compared through the Wilcoxon rank-sum test. We evaluated between sample diversity using the β-diversity with the unweighted UniFrac metric [23]. The unweighted UniFrac distance between a pair of samples m and k is defined as $U_{mk} = \frac{unique}{observed}$, where *unique* and *observed* represent the the unique and the total branch length in sample m or sample k. The β-diversities were visualized by the non-metric multidimensional scaling analysis (nMDS).

We used Spearman's correlation coefficients and proportionality between taxa to measure the taxa-taxa relationships. We excluded taxa with an excessive number of zeros (more than 90%) across all real samples. Then the pairwise Spearman's correlation coefficients or the "goodness-of-fit proportionality" statistic were calculated among all the remaining taxa.

Online resource for MB-GAN

We used Keras [32] with the Tensorflow [33] backend to implement the MB-GAN model. We provided the source codes, example output datasets and a Jupyter Notebook in GitHub as an online resource (https://github.com/zhanxw/MB-GAN). These include the trained models for the generator network and they can facilitate reproducing the results reported in this manuscript. They can also be customized to simulate new datasets for future microbiome studies. The codes are licensed under GNU General Public License v3.0.

Availability of source code and requirements

- Project name: MB-GAN
- Project home page: https://github.com/zhanxw/MB-GAN
- Operating system(s): Linux
- Programming language: Python (version 3.6.8), R (version 3.6.0)
- Other requirements: Tensorflow (version 1.14.0), Keras (version 2.2.4)

- License: GNU General Public License v3.0

Availability of supporting data and materials

The Python code to implement MB-GAN, the R code for NorTA and metaSPARSim simulation, and the codes to reproduce all the figures and tables are openly available in the Github repository https://github.com/zhanxw/MB-GAN.

List of abbreviations

EM: Earth Mover; GAN: Generative adversarial network; IBD: inflammatory bowel disease; MB-GAN: Microbiome generative adversarial network; MSE: Mean square error; MWAS: Metagenome-wide association studies; nMDS: Non-metric multidimensional scaling; NorTA: Normal-To-Anything; OTU: Operational taxonomic unit; WGAN: Wasserstein generative adversarial network; WGAN-GP: Wasserstein generative adversarial network with gradient penalty; ziNB: zero-inflated negative binomial.

Ethic

Not applicable.

Consent for publication

Not applicable.

Competing Interests

No competing interest reported by any author.

Funding

This work was supported by the National Institutes of Health [5P30CA142543, 5R01GM126479, 5R01HG008983].

Author's contributions

R.R and S.J performed the experiment. L.X, G.X, Y.X, D.J.L. and Q.L. provided resources and helpful discussions. R.R., S.J. and X.Z. designed the experiment, performed data analysis, wrote the software, and wrote the manuscript.

Acknowledgments

We thank Jessie Norris for her comments on the manuscript.

References

1. Nielsen R, Paul JS, Albrechtsen A, Song YS. Genotype and SNP calling from next-generation sequencing data. Nature Reviews Genetics 2011;12(6):443.
2. Zeller G, Tap J, Voigt AY, Sunagawa S, Kultima JR, Costea PI, et al. Potential of fecal microbiota for early-stage detection of colorectal cancer. Molecular systems biology 2014;10(11).
3. Qin J, Li Y, Cai Z, Li S, Zhu J, Zhang F, et al. A metagenome-wide association study of gut microbiota in type 2 diabetes. Nature 2012;490(7418):55.
4. Castro-Nallar E, Bendall ML, Pérez-Losada M, Sabuncyan S, Severance EG, Dickerson FB, et al. Composition, taxonomy and functional diversity of the oropharynx microbiome in individuals with schizophrenia and controls. PeerJ 2015;3:e1140.
5. Lee C, Lee S, Park T. A comparison study of statistical methods for the analysis metagenome data. In: 2017 IEEE International Conference on Bioinformatics and Biomedicine (BIBM) IEEE; 2017. p. 1777–1781.
6. Jiang S, Xiao G, Koh A, Kim J, Li Q, Zhan X. A Bayesian zero-inflated negative binomial regression model for the integrative analysis of microbiome data. Biostatistics (Oxford, England) 2019;.
7. Robinson MD, McCarthy DJ, Smyth GK. edgeR: a Bioconductor package for differential expression analysis of digital gene expression data. Bioinformatics 2010;26(1):139–140.
8. Li H. Microbiome, metagenomics, and high-dimensional compositional data analysis. Annual Review of Statistics and Its Application 2015;2:73–94.
9. Faust K, Sathirapongsasuti JF, Izard J, Segata N, Gevers D, Raes J, et al. Microbial co-occurrence relationships in the human microbiome. PLoS computational biology 2012;8(7):e1002606.
10. Layeghifard M, Hwang DM, Guttman DS. Disentangling interactions in the microbiome: a network perspective. Trends in microbiology 2017;25(3):217–228.
11. Li Z, Lee K, Karagas MR, Madan JC, Hoen AG, O'malley AJ, et al. Conditional regression based on a multivariate zero-inflated logistic-normal model for microbiome relative abundance data. Statistics in biosciences 2018;10(3):587–608.
12. Cario MC, Nelson BL. Modeling and generating random vectors with arbitrary marginal distributions and correlation matrix. Citeseer; 1997.
13. Goodfellow I, Pouget-Abadie J, Mirza M, Xu B, Warde-Farley D, Ozair S, et al. Generative adversarial nets. In: Advances in neural information processing systems; 2014. p. 2672–2680.
14. Kingma DP, Welling M. Auto-encoding variational bayes. arXiv preprint arXiv:13126114 2013;.
15. Radford A, Metz L, Chintala S. Unsupervised representation learning with deep convolutional generative adversarial networks. arXiv preprint arXiv:151106434 2015;.
16. Liang X, Hu Z, Zhang H, Gan C, Xing EP. Recurrent topic-transition gan for visual paragraph generation. In: Proceedings of the IEEE International Conference on Computer Vision; 2017. p. 3362–3371.
17. Donahue C, McAuley J, Puckette M. Adversarial audio synthesis. arXiv preprint arXiv:180204208 2018;.
18. Sidhom JW, Larman HB, Pardoll DM, Baras AS. DeepTCR: a deep learning framework for revealing structural concepts within TCR Repertoire. bioRxiv 2018;p. 464107.
19. Mahapatra D, Bozorgtabar B, Garnavi R. Image super-

resolution using progressive generative adversarial networks for medical image analysis. Computerized Medical Imaging and Graphics 2019;71:30–39.

20. Nielsen HB, Almeida M, Juncker AS, Rasmussen S, Li J, Sunagawa S, et al. Identification and assembly of genomes and genetic elements in complex metagenomic samples without using reference genomes. Nature biotechnology 2014;32(8):822.
21. Pasolli E, Schiffer L, Manghi P, Renson A, Obenchain V, Truong DT, et al. Accessible, curated metagenomic data through ExperimentHub. Nature methods 2017;14(11):1023.
22. Patuzzi I, Baruzzo G, Losasso C, Ricci A, Di Camillo B. metaSPARSim: a 16S rRNA gene sequencing count data simulator. BMC Bioinformatics 2019;20(9):1–13.
23. Lozupone C, Knight R. UniFrac: a new phylogenetic method for comparing microbial communities. Applied and Environmental Microbiology 2005;71(12):8228–8235.
24. Lovell D, Pawlowsky-Glahn V, Egozcue JJ, Marguerat S, Bähler J. Proportionality: a valid alternative to correlation for relative data. PLoS Computational Biology 2015;11(3).
25. Zhao N, Chen J, Carroll IM, Ringel-Kulka T, Epstein MP, Zhou H, et al. Testing in microbiome-profiling studies with MiRKAT, the microbiome regression-based kernel association test. The American Journal of Human Genetics 2015;96(5):797–807.
26. Gulrajani I, Ahmed F, Arjovsky M, Dumoulin V, Courville AC. Improved training of Wasserstein GANs. In: Advances in neural information processing systems; 2017. p. 5767–5777.
27. Lucic M, Kurach K, Michalski M, Gelly S, Bousquet O. Are gans created equal? a large-scale study. In: Advances in neural information processing systems; 2018. p. 700–709.
28. Arjovsky M, Chintala S, Bottou L. Wasserstein GAN. arXiv preprint arXiv:170107875 2017;.
29. Evans SN, Matsen FA. The phylogenetic Kantorovich–Rubinstein metric for environmental sequence samples. Journal of the Royal Statistical Society: Series B (Statistical Methodology) 2012;74(3):569–592.
30. Kurtz ZD, Müller CL, Miraldi ER, Littman DR, Blaser MJ, Bonneau RA. Sparse and compositionally robust inference of microbial ecological networks. PLoS computational biology 2015;11(5):e1004226.
31. Yoon G, Gaynanova I, Müller CL. Microbial networks in SPRING-Semi-parametric rank-based correlation and partial correlation estimation for quantitative microbiome data. Frontiers in Genetics 2019;10.
32. Chollet F, et al., Keras; 2015. https://keras.io.
33. Abadi M, Barham P, Chen J, Chen Z, Davis A, Dean J, et al. Tensorflow: A system for large-scale machine learning. In: 12th {USENIX} Symposium on Operating Systems Design and Implementation ({OSDI} 16); 2016. p. 265–283.

Click here to access/download
Supplementary Material
mbgan_supplement.pdf

Response letter

Click here to access/download
Supplementary Material
response_letter_XZ.pdf

Personal Cover

Click here to access/download;Personal Cover;Cover letter for revision.pdf

UTSouthwestern
Medical Center

Xiaowei Zhan, Ph.D.
Assistant Professor
Quantitative Biomedical Research Center
Department of Population and Data Sciences
Center for the Genetics of Host Defense
University of Texas Southwestern Medical Center
Dallas, TX 75390

December 15, 2020

Dear Editor:

We are submitting the revised manuscript “MB-GAN: Microbiome simulation via Generative Adversarial Network” by Rong et al., which we would like to be considered for publication in GigaScience.

We thank the comments by reviewers and editor for their detailed and constructive comments. As the manuscript requires “minor revision”, we have addressed all comments. Specifically, we verified that these aspects are significantly improved:

(1) The revised sentences in the main texts are highlighted in blue;

(2) All figures are in correct order to ease the readers;

(3) Simulation codes and documentation in GitHub repository enables reproduction of this work.

We hope that this revision can fully resolve the comments and this work can be considered for publication. Thank you.

Sincerely,

Xiaowei Zhan

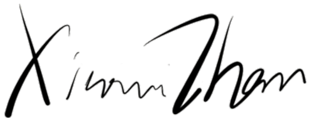

Supplement: giab005_GIGA-D-20-00286_Revision_1 [file giab005_giga-d-20-00286_revision_1.pdf]
