# Supplementary material for: MB-GAN: Microbiome Simulation via Generative Adversarial Network
Source: Gigascience. 2021 Feb 5;10(2):giab005. doi: 10.1093/gigascience/giab005 (PMC7931821; doi:10.1093/gigascience/giab005)
Supplement: giab005_Supplemental_File [file giab005_supplemental_file.pdf]

GigaScience, 2017, 1–20

doi: xx.xxxx/xxxx
Manuscript in Preparation
Research

RESEARCH

Supplement to: MB-GAN: Microbiome Simulation via Generative Adversarial Network

Ruichen Rong[1,†], Shuang Jiang[1,2,†], Lin Xu[1], Guanghua Xiao[1], Yang Xie[1], Dajiang J. Liu[3], Qiwei Li[4] and Xiaowei Zhan[1,*]

[1]University of Texas Southwestern Medical Center, Dallas, Texas, 75390, USA and [2]Southern Methodist University, Dallas, TX 75275, USA and [3]Pennsylvania State University, Hershey, Pennsylvania, 17033, USA and [4]The University of Texas at Dallas, Dallas, Texas, 75080, USA

*Xiaowei.Zhan@utsouthwestern.edu
†Contributed equally.

Compiled on: November 23, 2020.
Draft manuscript prepared by the author.

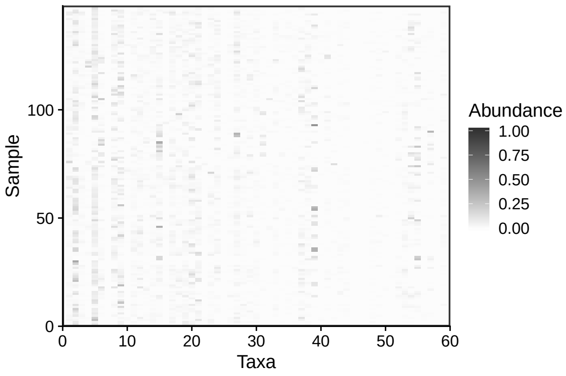

(a) Real data of the case group

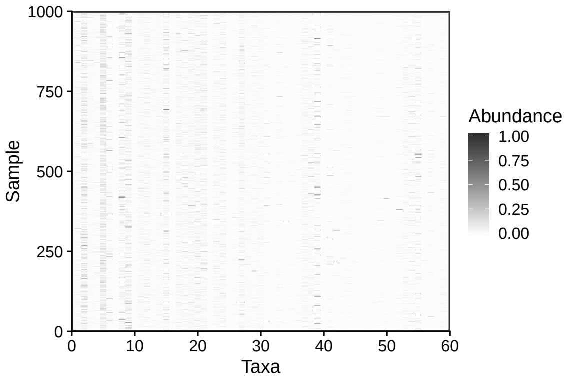

(b) MB-GAN simulated data for the case group

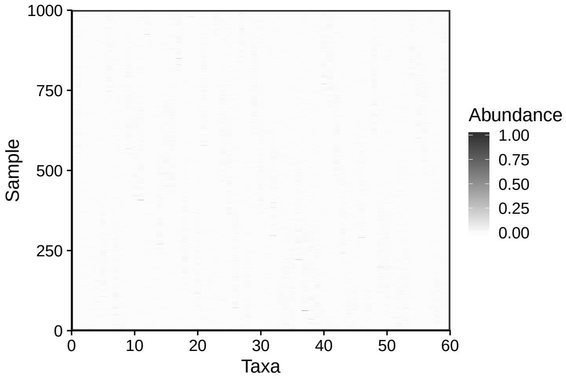

(c) Normal-To-Anything simulated data for the case group

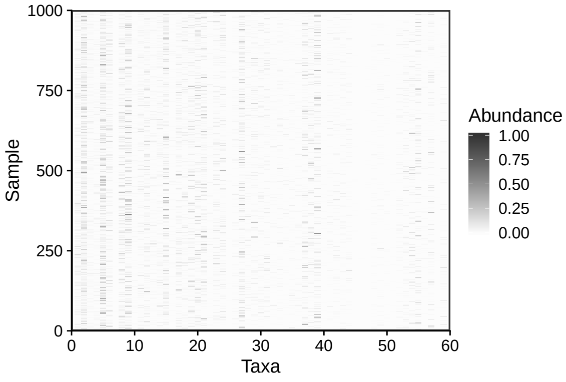

(d) metaSPARSim simulated data for the case group

Figure S1. Heatmaps of abundances of the 60 most abundant taxa (extracted from the real data) from (a) the 148 samples in the case group of the real data, (b) 1,000 simulated MB-GAN case samples after 20,000 iterations, (c) 1,000 simulated Normal-To-Anything case samples, and (d) 1,000 simulated metaSPARSim case samples

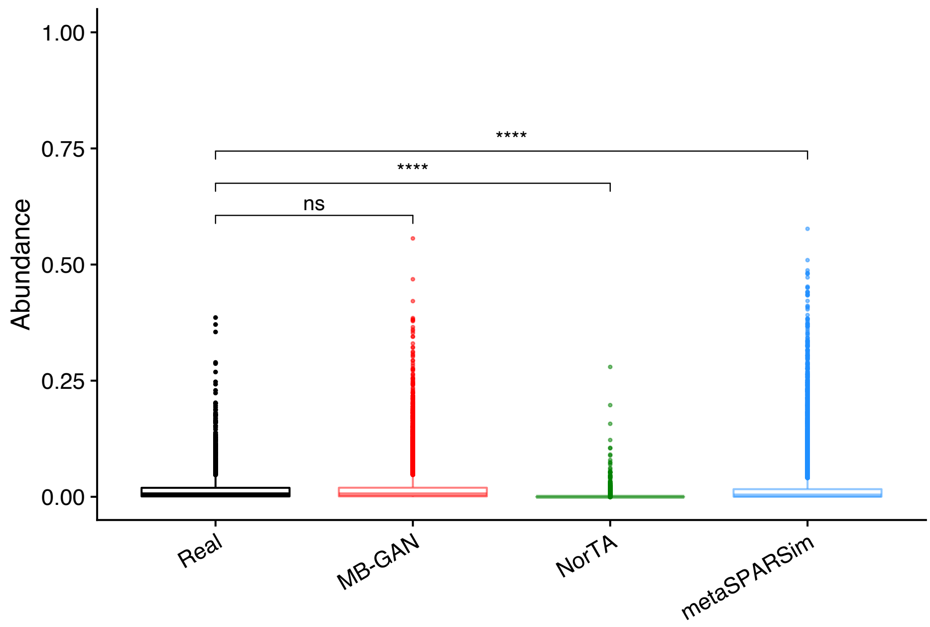

(a) Box plots of the abundances of taxa with less than 10% of zeros

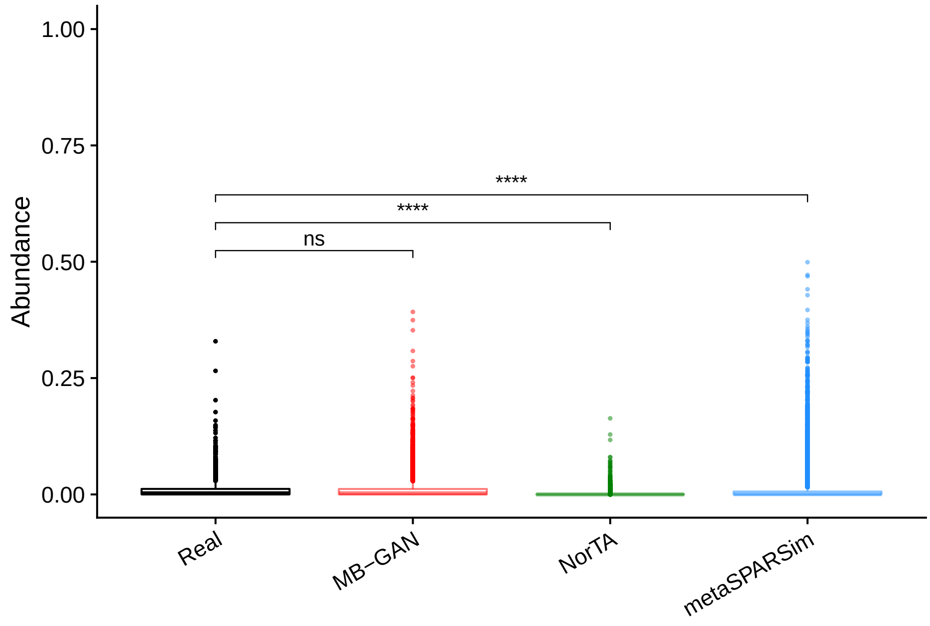

(b) Box plots of the abundances of taxa with 10%-20% of zeros

Figure S2. Case group: Box plots of the abundances of taxa with less than 10% of zeros (a), 10%-20% of zeros (b) in the real data of the case group. Wilcoxon rank-sum test is applied to compare the real and the simulated abundances by MB-GAN (p-value > 0.05), Normal-To-Anything (NorTA) (p-value < 0.0001), and metaSPARSim (p-value < 0.0001).
Notations: ns – not significant; **** – p-value < 0.0001.

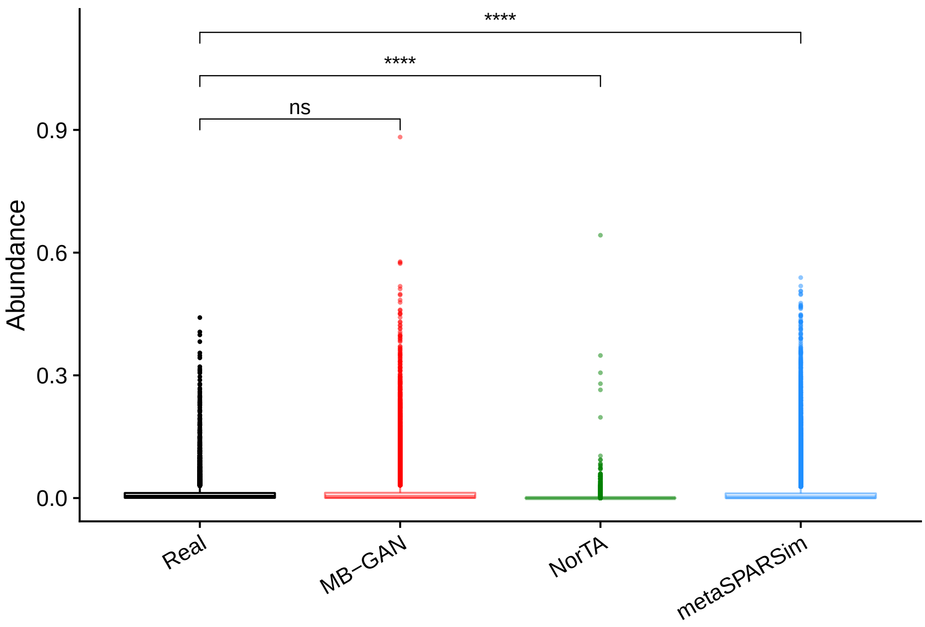

(a) Box plots of the abundances of taxa with less than 10% of zeros

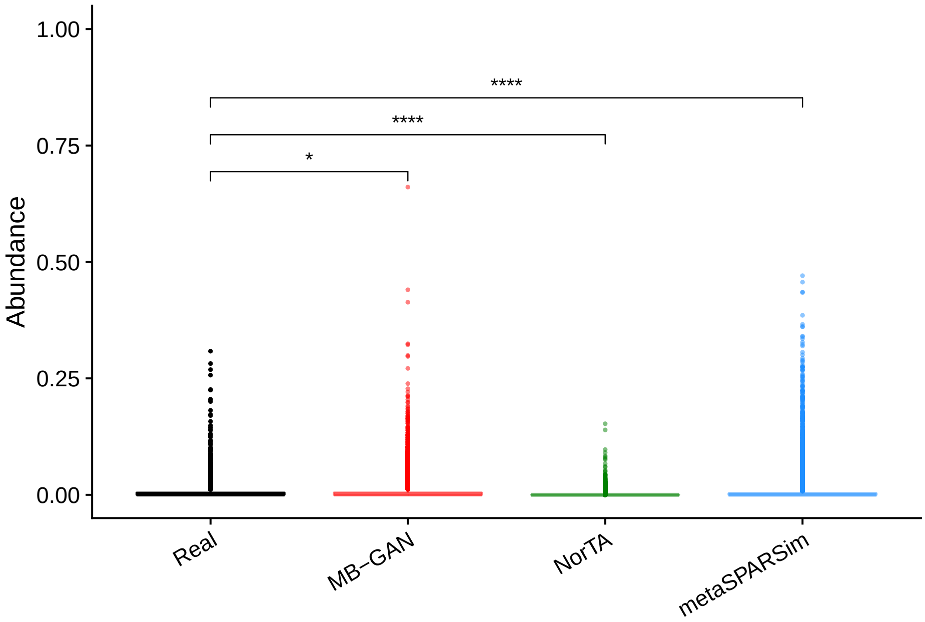

(b) Box plots of the abundances of taxa with 10%-20% of zeros

Figure S3. Control group: Box plots of the abundances of taxa with less than 10% of zeros (a), 10%-20% of zeros (b) in the real data of the control group. Wilcoxon rank-sum test is applied to compare the real and the simulated abundances by MB-GAN (p-value > 0.01), Normal-To-Anything (NorTA) (p-value < 0.0001), and metaSPARSim (p-value < 0.0001).
Notations: ns – not significant; * – p-value between 0.01 – 0.05; **** – p-value < 0.0001.

Evaluating MB-GAN on a Smaller Microbiome Dataset

To comprehensively examine the performance of MB-GAN, we performed a similar analysis on a gut microbiome dataset with smaller sample size compared to Nielsen et al. [1]. Again, we focused on how different simulation methods captured the sample-level properties and the taxa-taxa relationships on the smaller dataset. Results from the new data confirmed the better performance of MB-GAN over Normal-To-Anything (NorTA) and metaSPARSim.

Data Description

We extracted 111 sequenced shotgun metagenomic samples from the gut microbiome study published by Zeller et al. [2]. The original dataset contains the measurement of $1,585$ taxa (from species to phylum level) on 52 colorectal cancer (CRC) patients and 59 healthy controls. The original metagenomic sequence data from the fecal samples are available in the European Nucleotide Archive Database (accession number ERP005534). The phylogenetic tree accompanied to the original sequencing data was extracted from the R package `curatedMetagenomicData`.

Analyses

We simulated microbiome abundances using MB-GAN for the CRC and the control group respectively. We adapted the same default setting used for MB-GAN simulation described in the main text (see more details in Method section), and generated 1,000 MB-GAN samples for each group. Meanwhile, we implemented the other two alternative methods, NorTA and metaSPARSim, to obtain $1,000$ samples for each group separately.

By comparing the performances of the three generators, we demonstrated the high-fidelity of MB-GAN samples to the real data. We compared the simulated and observed abundances to examine the fidelity of the MB-GAN samples in different taxa abundance strata. In the CRC group, we considered (1) the taxa having less than 10% zeros across all the samples, and (2) the taxa having 10% $\sim$ 20% zeros across all the samples. We used the Wilcoxon rank-sum test to compare the observed and three types of simulated abundances. As shown in Figure S4 (a) the MB-GAN samples give p-values > 0.05 in both scenarios, whereas the other two types of simulated samples showed significantly different abundances (p-values < 0.0001). We performed the same analysis on the control group, and the results are shown in Figure S4 (b). The p-values are 0.65 for scenario (1) and (2) when testing the MB-GAN simulated abundances against the observed ones. The p-values from the NorTA and metaSPARSim simulated data are less than 0.0001 in both scenarios. The results confirmed that the MB-GAN performed better in capturing the distributions of those more abundant taxa.

Evaluation on sample-level properties

We compared the sample-level statistics calculated from the real data and the simulated data (by MB-GAN, NorTA, and metaSPARSim). The sample-level properties included sparsity, α-diversity, and β-diversity. As NorTA dropped the taxa with zeros across all samples in its simulation process, the comparisons focus on the species shared among the real data and the three simulators.

First, we evaluated sample sparsity, which is the proportion of zeros in a sample. Table S1 summarizes the results for the CRC and the control group. Again, the MB-GAN samples tended to have a higher sparsity than the real data, yet the other two methods underestimated the sparsity for both groups. We also noticed that the minimum of sparsity given by NorTA and metaSPARSim was generally higher than both the real and MB-GAN samples.

(a) Sparsity of the CRC group

	min	median	max
Real data	0.511	0.760	0.863
MB-GAN	0.613	0.814	0.942
NorTA	0.624	0.742	0.829
metaSPARSim	0.670	0.712	0.778

(b) Sparsity of the control group

	min	median	max
Real data	0.599	0.736	0.852
MB-GAN	0.547	0.797	0.945
NorTA	0.623	0.734	0.810
metaSPARSim	0.658	0.708	0.760

Table S1. Sample sparsity calculated from four datasets (Real, MB-GAN, Normal-To-Anything (NorTA), and metaSPARSim) under (a) CRC or (b) control group.

Next, we evaluated the α-diversities of the three types of simulated datasets by comparing the Shannon indices. A better matching between the Shannon indices from the real and simulated data suggests that the simulator could capture the biodiversity observed in the real data. As shown by the box plots in Figure S5, the Shannon indices from MB-GAN samples matched consistently with the real data in both groups (p-value from the Wilcoxon rank-sum test > 0.05). However, the Shannon indices by NorTA samples were clearly larger than the real ones, and Shannon indices by metaSPARSim samples tended to be smaller than the real ones. The Wilcoxon rank-sum test yielded p-values less than

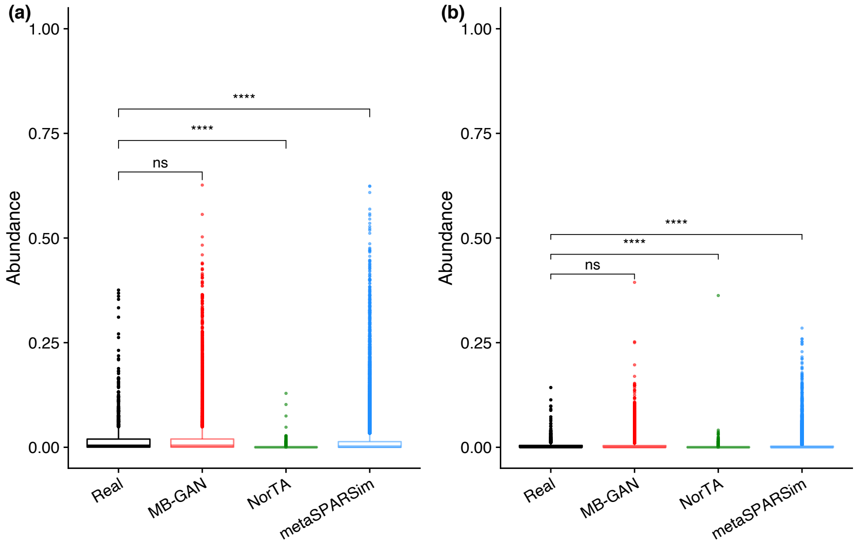

(a) Box plots comparing taxa abundance for the colorectal cancer group

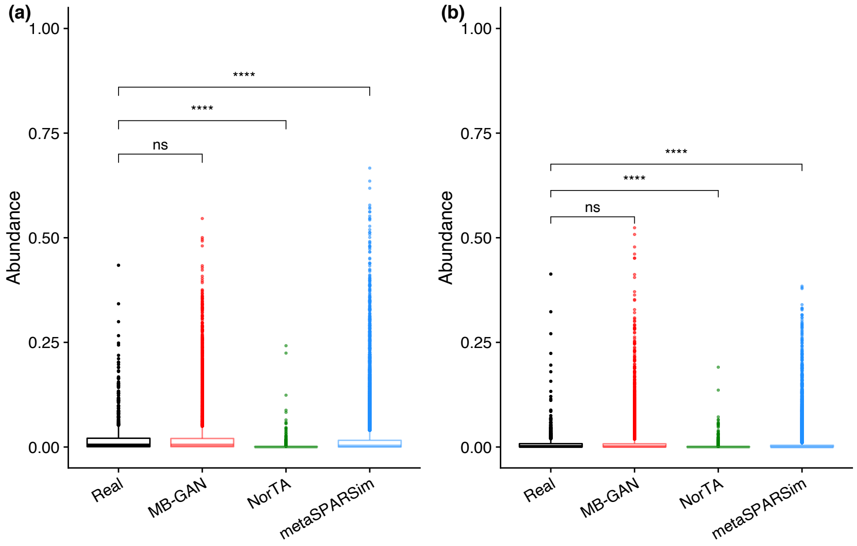

(b) Box plots comparing taxa abundance for the healthy control group

Figure S4. Box plots of the abundances of taxa with less than 10% of zeros (left), 10%-20% of zeros (right) in the real data [2]. Wilcoxon rank-sum test is applied to compare the simulated abundances by MB-GAN, Normal-To-Anything, metaSPARSim, and the observed abundances. (ns: not significant; ****: p-value < 0.0001.)

0.0001 for both simulators in the CRC and the control groups. In all, our results suggest that the MB-GAN simulated data can better mimic the real microbiome abundances with respect to α-diversity.

Finally, we evaluated the β-diversity of the simulated data from MB-GAN, NorTA, and metaSPARSim. The comparison was based on non-metric multidimensional scaling (nMDS) analysis using the UniFrac distance to incorporate the phylogenetic information. Figure S6 (a) and (b) visualize the results for the CRC and the control group, respectively. In both scenarios, the MB-GAN samples shared similar patterns with the real data in the two-dimensional plot. The NorTA and the metaSPARSim samples showed unique patterns in the nMDS analysis. For example, the nMDS plot by NorTA samples demonstrate a circular pattern, while metaSPARSim samples are separated by several clear gaps in the plot. Interestingly, these patterns were also observed in the nMDS plot in the main text. We did not observe these patterns from the real data or the MB-GAN samples. Therefore, the nMDS analysis here showed that MB-GAN performed better than NorTA and metaSPARSim with respect to capturing the β-diversity from the real data.

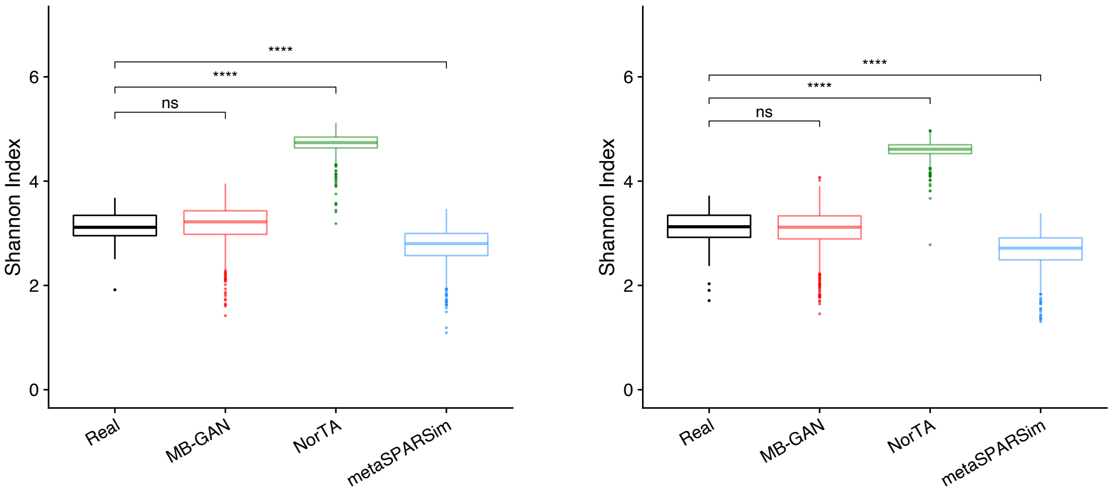

(a) Shannon index of the case group

(b) Shannon index of the control group

Figure S5. Box plots of Shannon index calculated from three datasets (Real, MB-GAN, Normal-To-Anything (NorTA), and metaSPARSim) under (a) CRC or (b) control group. (ns: not significant; ****: p-value < 0.0001.)

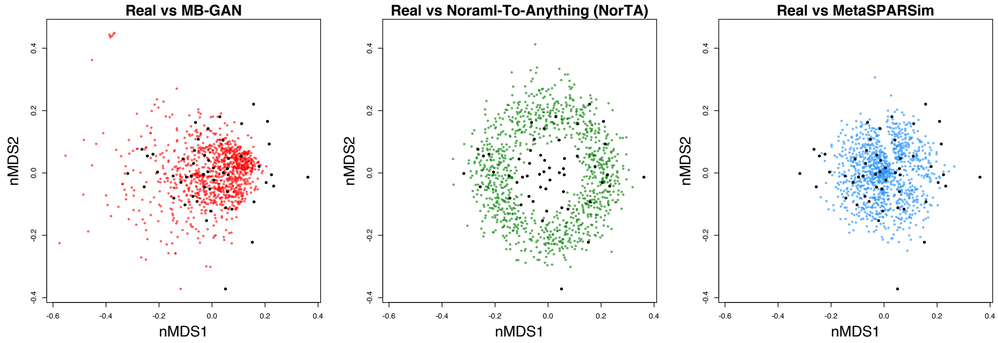

(a) nMDS of the CRC group

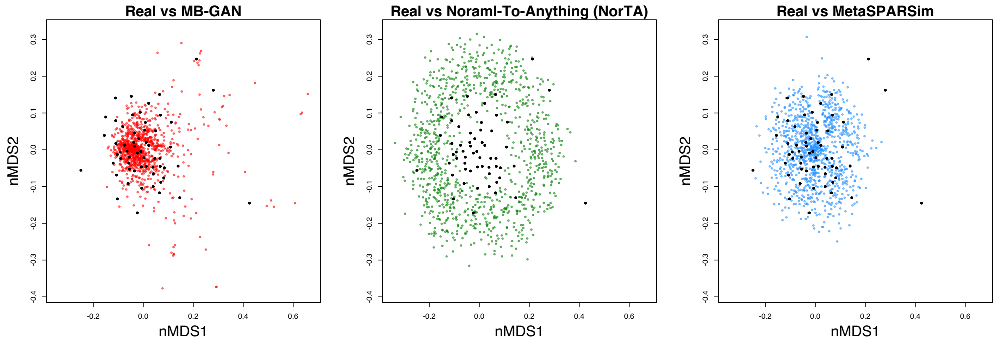

(b) nMDS of the control group

Figure S6. β-diversity visualization using non-metric multidimensional scaling (nMDS) for (a) the CRC and (b) the control group. For samples from the real data, the MB-GAN, the Normal-To-Anything (NorTA), and the metaSPARSim simulated data, the two-dimensional nMDS values were calculated using the UniFrac metric.

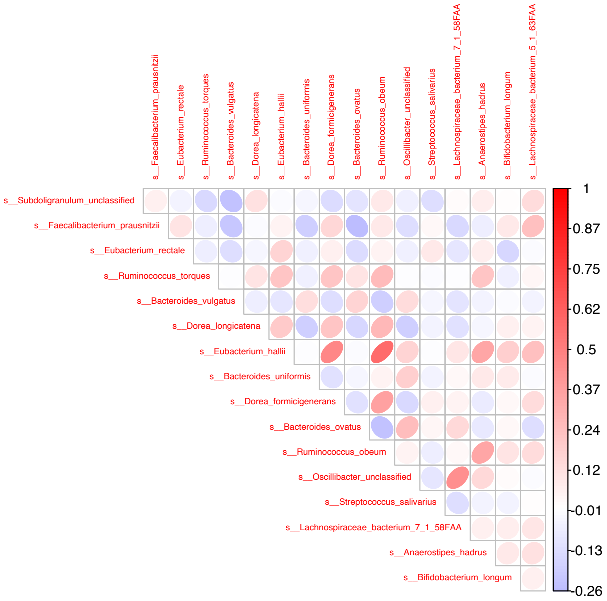

Figure S7. Correlogram of Spearman's correlation coefficients calculated from the top 10% most abundant species in the case group. Spearman's correlations are calculated based on the real data with names of the species shown.

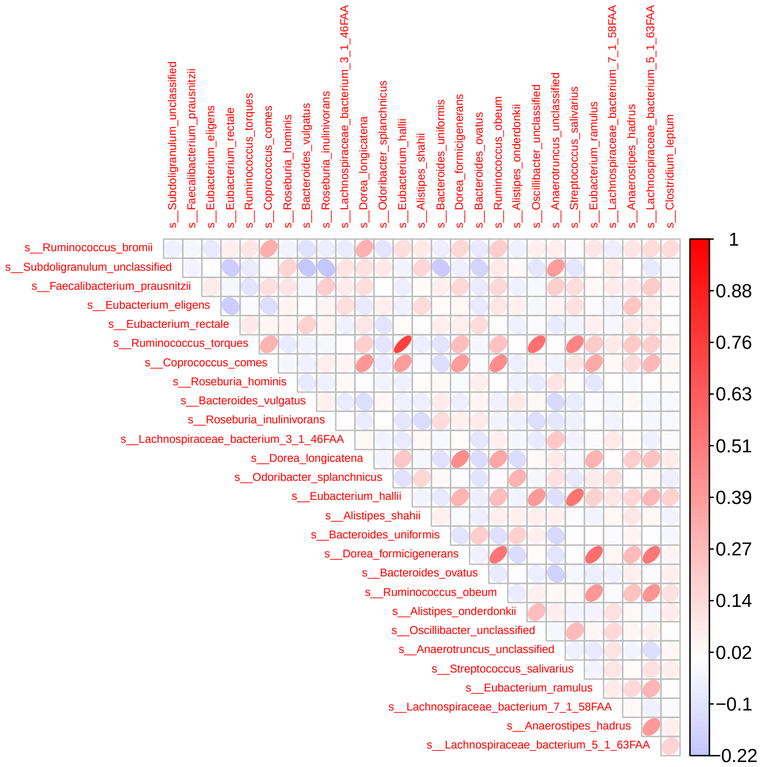

Figure S8. Correlogram of Spearman's correlation coefficients calculated from the top 10% most abundant species in the control group. Spearman's correlations are calculated based on the real data with names of the species shown.

Evaluation on taxa-taxa relationships

We measured the second order characteristics of the simulated data using the Spearman's correlation coefficients and proportionality. The scatter plots in Figure S9 (a) and (b) visualize the Spearman's correlations calculated from the real and the simulated data, using the top 10% most abundant species from the CRC and the control group, respectively. The R^2 and mean square error (MSE) are included in the plot. We observed that the correlation structure from MB-GAN samples resembled that of the real data for both groups. Whereas for the other two methods, the correlation structures seemed to be quite different from the real data, as shown in the second and third subfigure in Figure S9 (a), (b). The results given by metaSPARSim suggested that the simulated taxa had weak association measured by the Spearman's correlation coefficient.

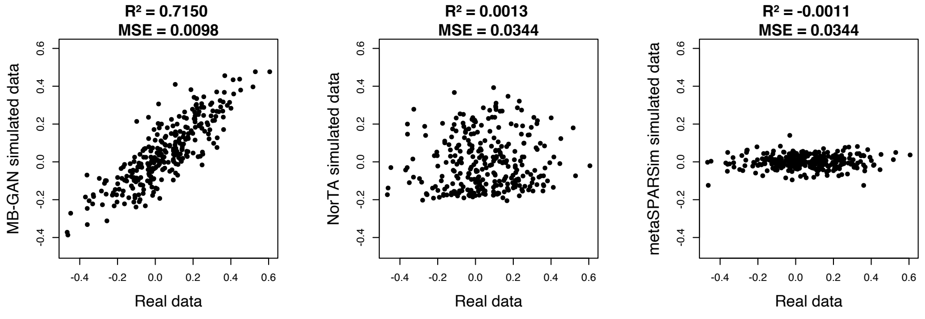

(a) CRC group: Spearman correlation coefficients between the real and the three types of simulated data using the top 10% most abundant species from the real data (from the CRC group)

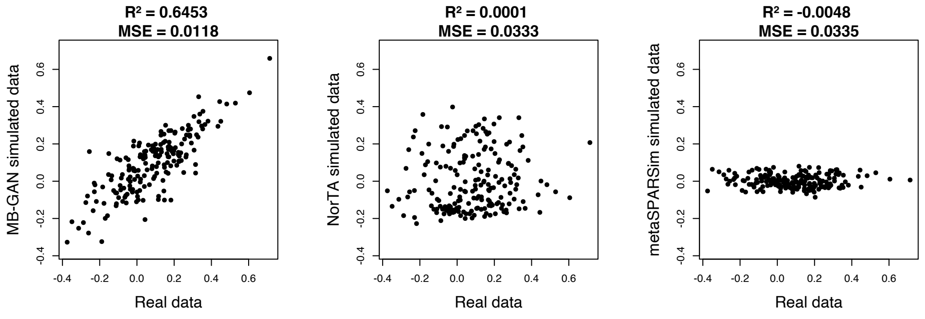

(b) Control group: Spearman correlation coefficients between the real and the three types of simulated data using the top 10% most abundant species from the real data (from the control group)

Figure S9. Scatter plot of the Spearman correlation coefficients between the real and the simulated data by MB-GAN, Normal-To-Anything (NorTA), and metaSPARSim. Resulted are calculated based on the top 10% most abundant species from the real data of the (a) CRC and the (b) control group, respectively.

MB-GAN performed better in capturing the taxa-taxa relationships measured by Spearman's correlation. The correlograms in Figure S12 (a) present four Spearman's correlation matrices. A blue ellipse represents a negative correlation, while a red one suggests a positive correlation. The darker the color, or the shorter the ellipse's minor axis, the stronger the correlation between the corresponding taxa pair. Clearly, MB-GAN captured the overall pattern observed in the correlogram of the real data, and it preserved the relatively strong associations as well. Meanwhile, all the three simulators tended to show weaker associations compared to the real data, especially for metaSPARSim. Further, we observed a clear disparity between the correlograms by NorTA and the real data. The above conclusions also held for the control group, as similar results are shown in Figure S12 (b). In all, Figure S9 and S12 illustrated the overall better performance of MB-GAN over NorTA and metaSPARSim with respected to capturing the taxa-taxa relationships measured by Spearman's correlation.

Both the MB-GAN and metaSPARSim samples matched the proportionality well compared to the real data. The scatter plots in Figure S14 (a) and (b) visualize the proportionality of the real and three types of simulated data, using the top 10% most abundant species from the CRC and the control group, respectively. The correlation coefficient (R^2) and mean square

error (MSE) are provided in the plot. The metaSPARSim yielded slightly smaller MSE than the MB-GAN in both cases, and both MB-GAN and metaSPARSim performed much better than NorTA. Results from the real data suggested a lot of larger values of proportionality, while the three simulators, especially NorTA and metaSPARSim, generated species with much smaller proportionality. In general, MB-GAN and metaSPARSim performed well in preserving the proportionality in the real data. However, as shown in the previous section, MB-GAN outperformed metaSPARSim by maintaining a valid representation on the sample-level properties of the real data.

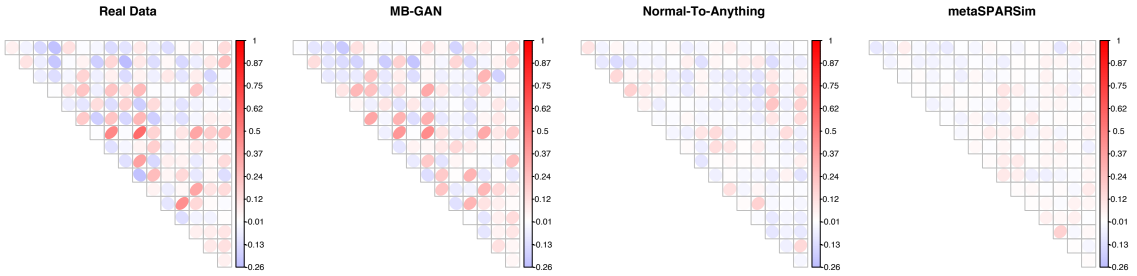

(a) Correlograms of Spearman correlation coefficients of the top 10% most abundant taxa

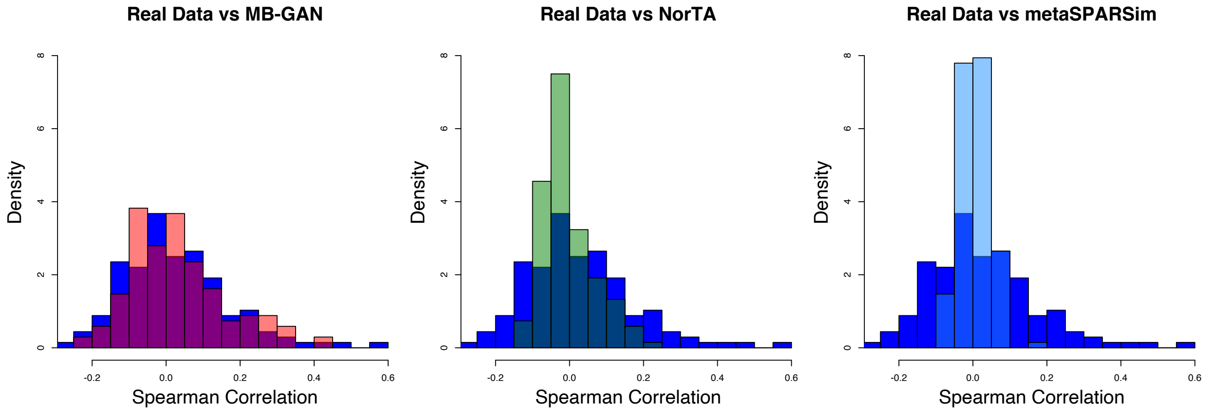

(b) Empirical distribution of Spearman correlation coefficients of the top 10% most abundant taxa

Figure S10. Case group: Comparison of correlograms and empirical distributions of the Spearman correlation coefficients calculated from the real data and the three types of simulated data. The correlation coefficients are calculated from the top 10% most abundant species in the case group.

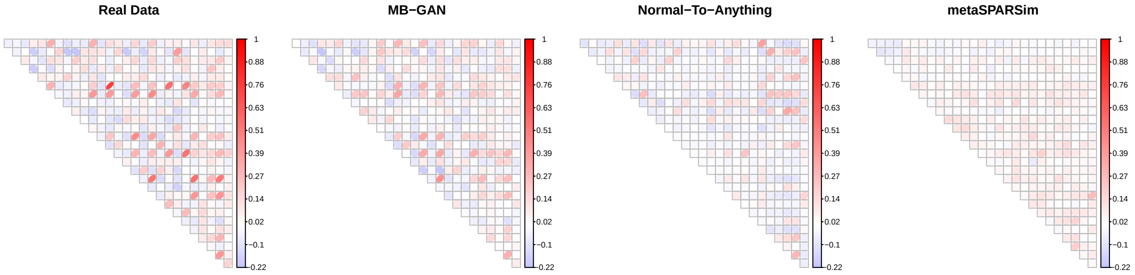

(a) Correlograms of Spearman correlation coefficients of the top 10% most abundant taxa

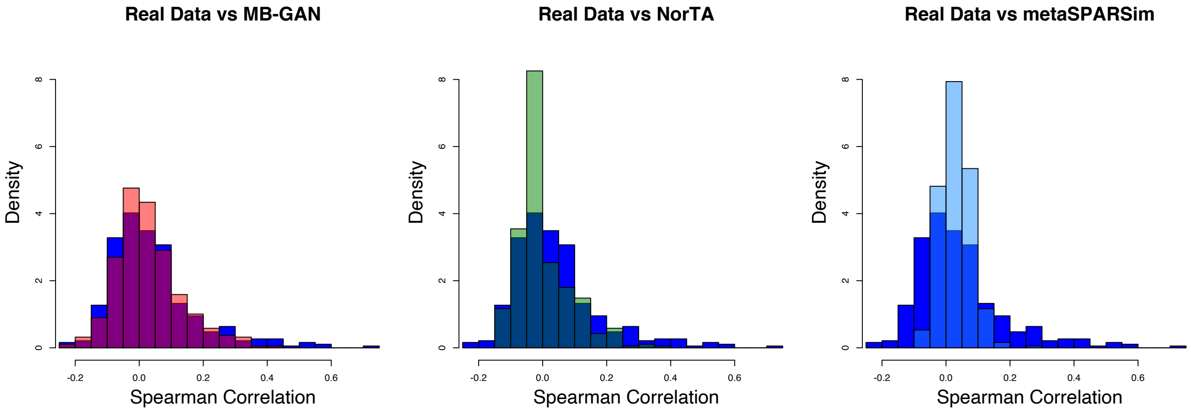

(b) Empirical distribution of Spearman correlation coefficients of the top 10% most abundant taxa

Figure S11. Control group: Comparison of correlograms and empirical distributions of the Spearman correlation coefficients calculated from the real data and the three types of simulated data. The correlation coefficients are calculated from the top 10% most abundant species in the control group.

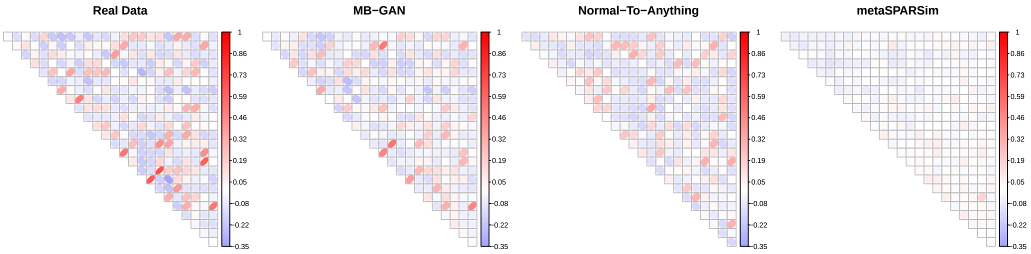

(a) CRC group: Correlograms of Spearman correlation coefficients of the top 10% most abundant taxa

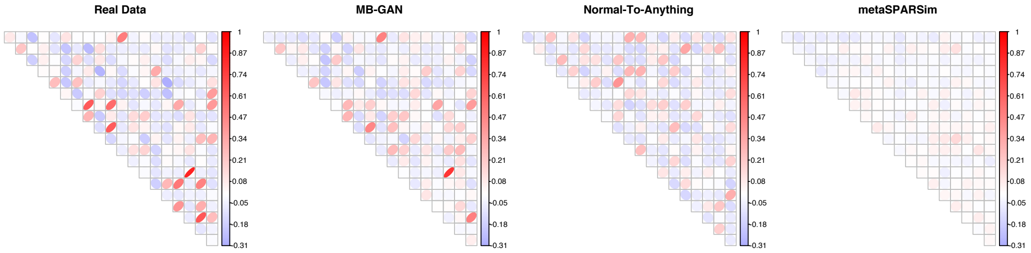

(b) Control group: Correlograms of Spearman correlation coefficients of the top 10% most abundant taxa

Figure S12. Comparison of correlograms of the Spearman correlation coefficients calculated from the real data [2] and the three types of simulated data. The correlation coefficients are calculated from the top 10% most abundant species in the the CRC and the control group, respectively.

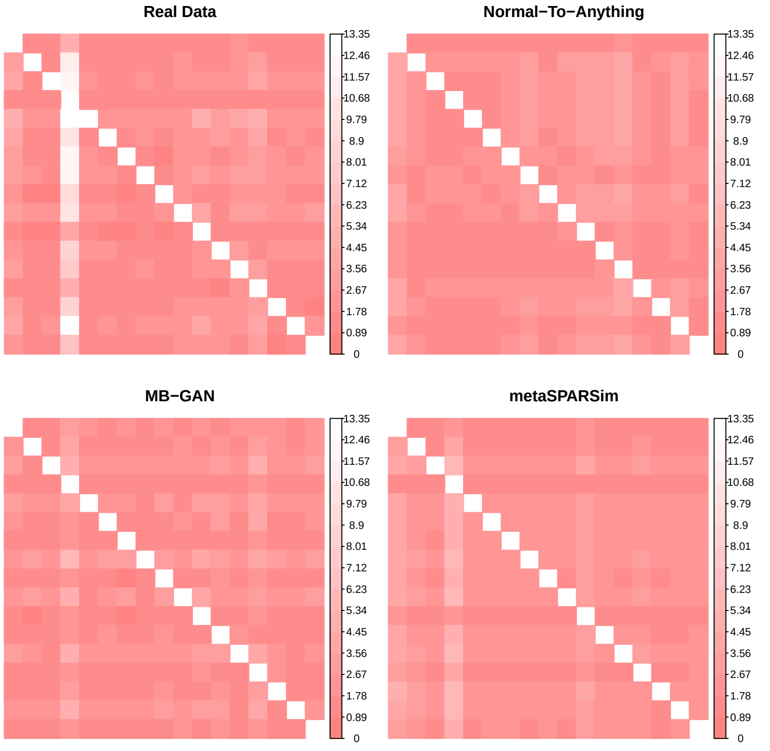

(a) Case group: Heatmaps of proportionality of different datasets based on the same top 10% most abundant taxa from the case group

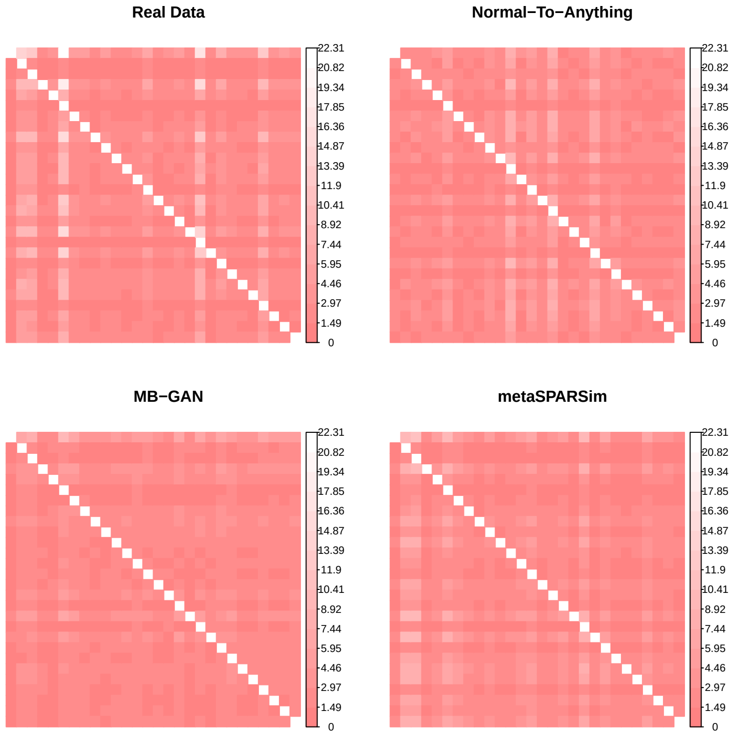

(b) Control group: Heatmaps of proportionality of different datasets based on the same top 10% most abundant taxa from the contrl group

Figure S13. Heatmaps showing the pattern of proportionality calculated from the top 10% most abundant taxa of real data, and proportionality of the simulated data by MB-GAN, Normal-To-Anything, and metaSPARSim, based on the same top abundant taxa. Results calculated from the case and the control groups are shown in (a) and (b), respectively.

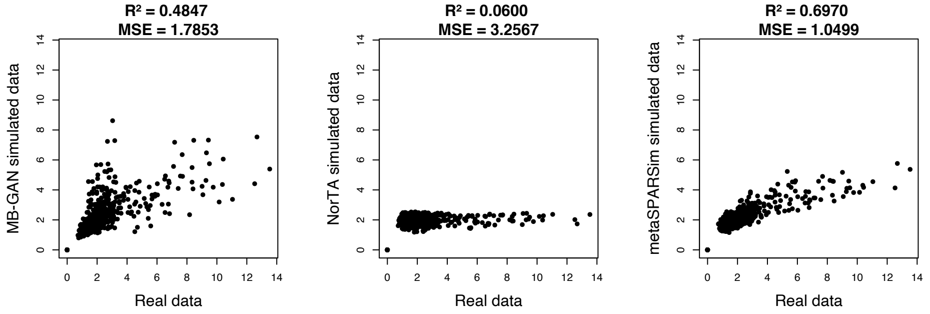

(a) CRC group: Proportionality between the real and three types of simulated data using the top 10% most abundant species from the real data

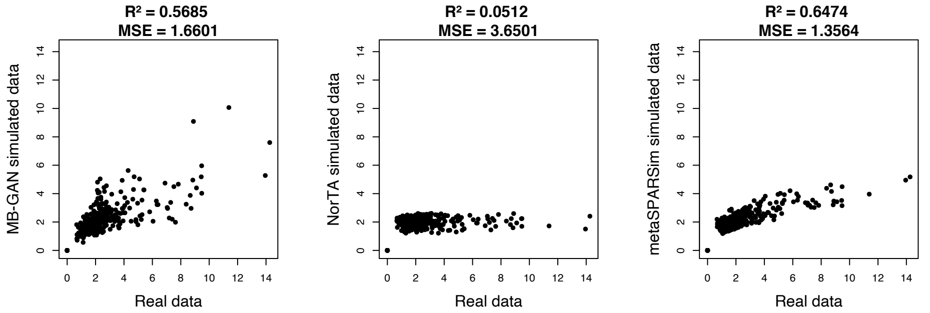

(b) Control group: Proportionality between the real and three types of simulated data using the top 10% most abundant species from the real data

Figure S14. Scatter plot of the "goodness-of-fit proportionality" statistic between the real and the simulated data by MB-GAN, Normal-To-Anything (NorTA), and metaSPARSim. Results are calculated based on the top 10% most abundant species from the real data [2] of the (a) CRC and the (b) control group, respectively.

Differential Abundance Analysis by MB-GAN

We carried out an exemplar differential abundance (DA) analysis based on two types of data, and evaluated the consistency between the results. The first type of data only consists of the original samples from the gut microbiome study [1] discussed in the manuscript. The second type mixed the original data with additional MB-GAN samples. We aimed at comparing the top differentially abundant taxa selected based on the two datasets. Our goal here was to examine whether adding the MB-GAN samples could reach the same conclusion as using only the real samples. If not, it need to be cautious when drawing conclusion based on microbiome datasets enlarged by MB-GAN samples.

We performed the DA analysis as follows. For the original data with $n_1 = 148$ IBD patients and $n_2 = 248$ controls, we implemented the Wilcoxon rank-sum test on each of the 719 species, and selected the 10 species with smallest p-values. The 10 species were ranked in the x-axis in Figure S15 below. Next, we randomly chose 200 MB-GAN simulated samples to mix into the case or the control groups of the real data, respectively. In total, we added 400 MB-GAN samples to the real data. Using the enlarged dataset, we computed the p-values for the 719 species again, and we extracted the ranks of p-values of the same 10 species on the new data. We repeated the above process 100 times. Each box in Figure S15 showed the ranks of the species' p-values across 100 replicates. The dashed line pinpointed the 10th rank.

The results showed that it needs to be cautious to draw conclusions based on the MB-GAN simulated samples, since we observed that several differentially abundant species detected based on the real data were totally missed after we added in the fake samples. The conclusion could be invalid if one intended to use MB-GAN samples as true samples to explore their biological functions. Hence, one must be cautious in designing the statistical experiments with the MB-GAN samples. Again, we suggest using MB-GAN as a model development tool for generating simulated data, instead of treating them as real data to enlarge existing sample sizes.

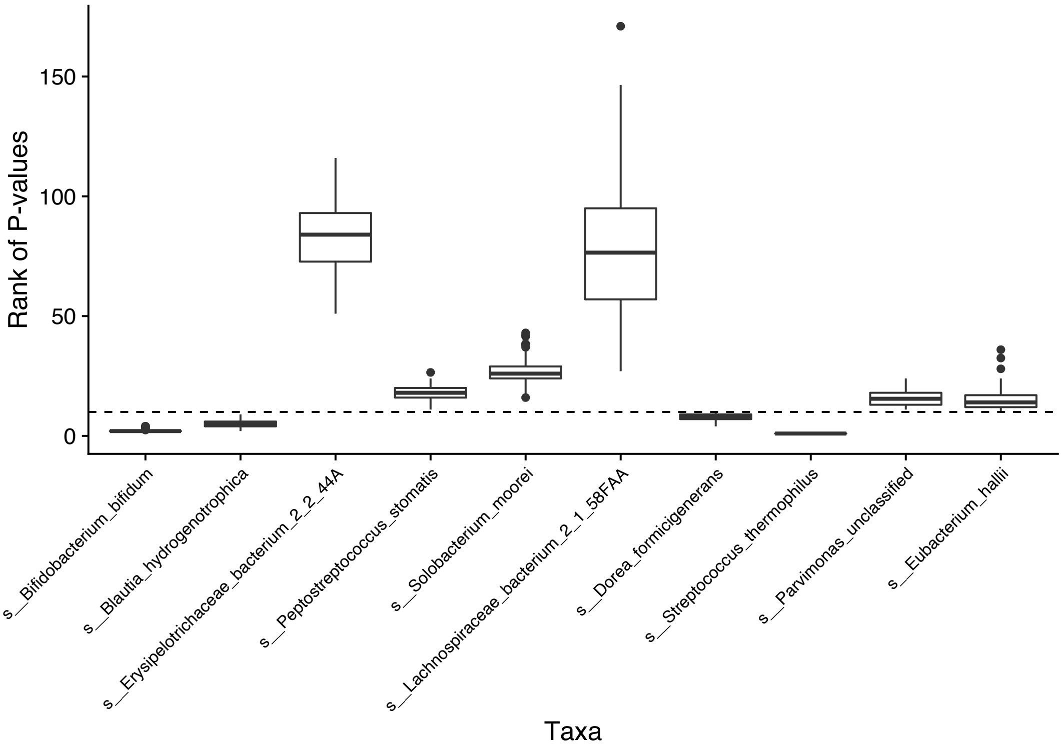

Figure S15. Box plot of the rank of p-values for the top 10 differentially abundant species selected based on the real data. Each box contains the ranked p-values from the Wilcoxon rank-sum test after adding in 400 MB-GAN samples for the corresponding species. The simulation was repeated for 100 times. The dashed line marks the rank at 10.

Using MB-GAN to Design the Simulation Study of Evaluating MiRKAT

Here, we provide a practical example that utilizes the MB-GAN samples to evaluate a statistical model proposed for metagenome-wide association studies (MWAS). The model, MiRKAT, is a regression-based kernel association test for microbiome data [3]. MiRKAT incorporates the information such as phylogenetic relationships among taxa, and samples' microbiome compositions in testing the association between microbial community profiles and outcomes. Hence, examining the model performance on simulated datasets that highly resemble the real data could provide better evaluations of mdoel performances. Here, we evaluated the statistical power of MiRKAT under different kernels to explore their performances on data that resembled the real microbiome sequencing outcomes.

We closely followed the model evaluation design proposed in Zhao et al. [3]. Specifically, we considered the "scenario 2" presentation in the original paper, where the continuous outcome of each sample was generated based on the top 10 abundant species in the data. We adapted the Equation (7) in Zhao et al. [3] to simulate the outcome variable, y_i, for each sample i. Equation (1) shows our data generating model.

$$y_i = 0.5X_i + \beta \text{scale}\left(\sum_{j \in A} \frac{Z_{i(j)}}{\bar{Z}_{(j)}}\right) + \epsilon_i, \quad i = 1, \ldots, n. \tag{1}$$

Here, X_i is an observed covariate for sample i, and ϵ_i is the error term. $Z_{i(j)}$ is the observed relative abundance of the jth taxon in the ith sample, and $\bar{Z}_{(j)}$ is the averaged abundance of jth taxon across all the samples. The set A consists of the indices of the top 10 abundant species. The "scale" function standardizes the taxa to have means of 0 and variances of 1.

We used the MB-GAN samples generated for the case group from Nielsen et al. [1] as the microbiome abundances (i.e., $Z_{i(j)}$'s in Model (1)). We generated $X_i \sim N(0, 1)$, $\epsilon_i \sim N(0, 1)$, and varied the effect size $\beta \in \{0, 0.25, 0.5, 0.75\}$. Following the implementation described in Zhao et al. [3], we used seven different kernels: K_w, K_u, K_{BC}, K_0, $K_{0.25}$, $K_{0.5}$, $K_{0.75}$, representing the MiRKAT results for the weighted UniFrac kernel, unweighted UniFrac kernel, Bray-Curtis kernel, and generalized UniFrac kernels with $\alpha = 0,\ 0.25, 0.5$, and 0.75, respectively. In addition, we included the optimal MiRKAT by considering all the seven kernels above. In total, we compared the performance of eight kernels. The sample size was fixed at $n = 100$. Under each β, we generated 2,000 replicated datasets to calculate either the Type-I error ($\beta = 0$) or the statistical power ($\beta \neq 0$) for each kernel.

The results based on MB-GAN samples are shown in Figure S16 below. Our results shared important similarities and interesting differences compared with the results in Zhao et al. [3]. We observed consistent findings for the best two and the worst kernels with respect to Type-I error and power. In particular, The Bray-Curtis kernel yielded the best performance, and the optimal kernel ranked the second. Results by the unweighted kernel showed the worst performance in both our simulation and in the original paper. However, for the rest kernels, the rankings with respect to their statistical power are different. Further, the results here suggested higher power as compared to the original study [3]. Therefore, we could see that difference strategies in simulating microbiome abundances could bias the estimation of performances of different methods, even the underlying statistical model (1) stayed the same. Hence, to evaluate the statistical models designed for MWAS, we recommend using MB-GAN for the design of simulation study.

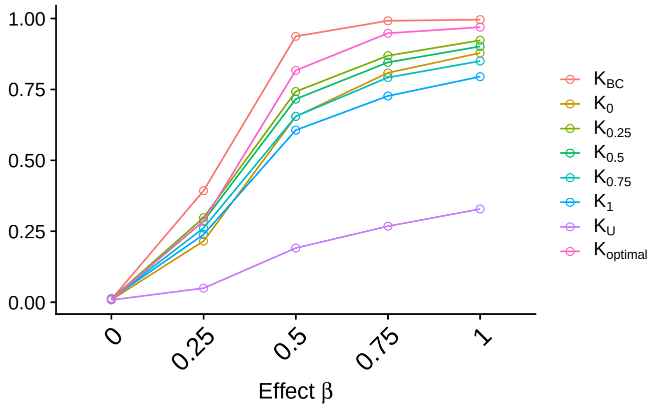

Figure S16. Type-I error and power of MiRKAT based on different kernels calculated from the MB-GAN simulated data. The eight kernel used here are: K_w, K_u, K_{BC}, K_0, $K_{0.25}$, $K_{0.5}$, $K_{0.75}$, representing MiRKAT results for the weighted UniFrac kernel, unweighted UniFrac kernel, Bray-Curtis kernel, and generalized UniFrac kernels with $\alpha = 0,\ 0.25, 0.5$, and 0.75, respectively. The Type-I error and power were calculated based on 2,000 replicates.

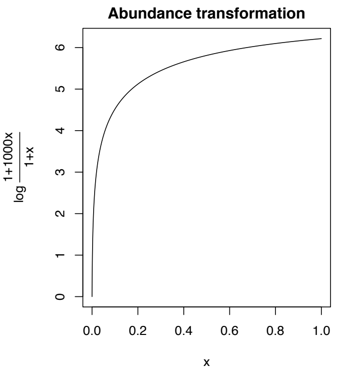

Figure S17. The phylogenetic transformation function. This function was used to transform the simulated microbiome abundances in the MB-GAN framework.

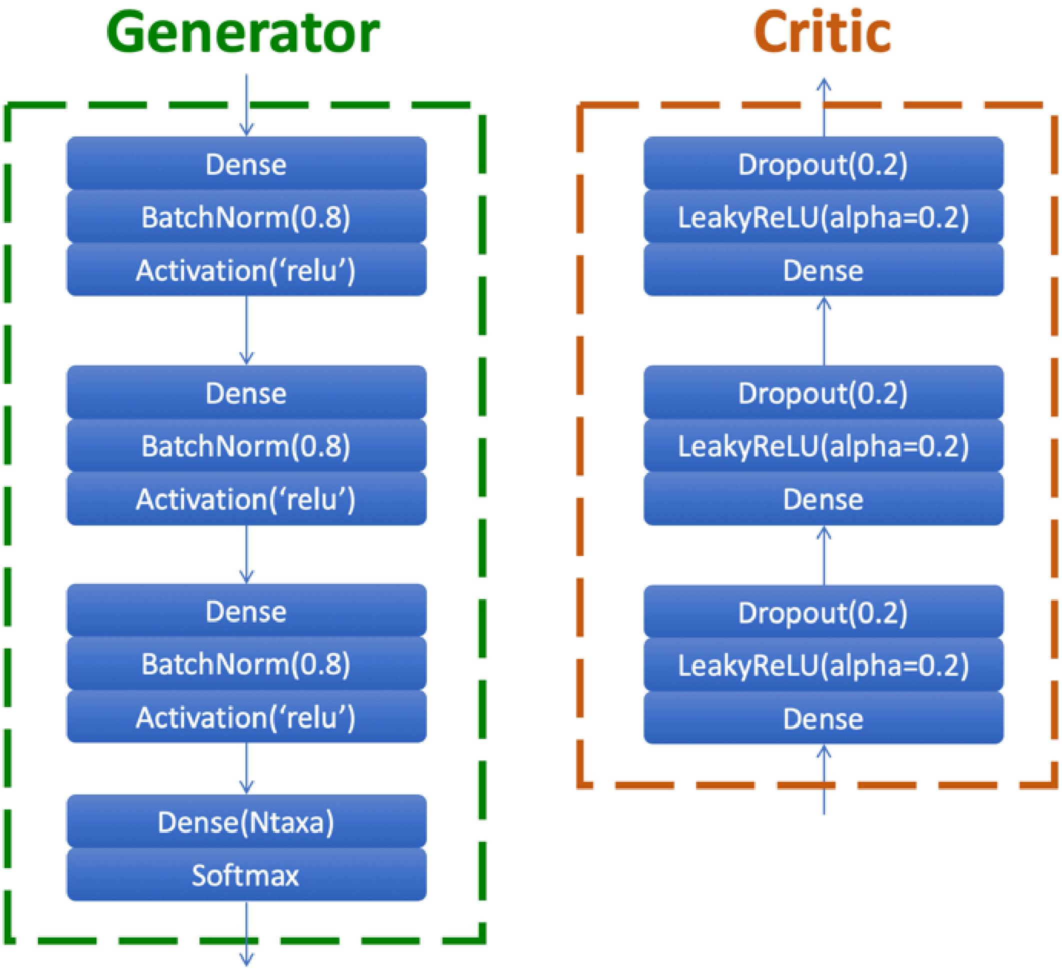

Figure S18. The architecture for the generator and critic model.
Notations:
Dense: a fully connected neural network layer;
BatchNorm(0.8): Batch normalization layer with momentum of moving mean and variance set to 0.8;
Activation('relu'): activation layer with ReLU activation function;
LeakyReLU(alpha = 0.2): a variation of ReLU activation layer and the activation function is $f(x) = x$ for $x \geq 0$, and $f(x) = \alpha \cdot x$ for $x < 0$;
Ntaxa: number of taxa;
Softmax: a softmax activation layer;
Dropout(0.2): a dropout layer with dropout rate set to 0.2.

References

1. Nielsen HB, Almeida M, Juncker AS, Rasmussen S, Li J, Sunagawa S, et al. Identification and assembly of genomes and genetic elements in complex metagenomic samples without using reference genomes. Nature biotechnology 2014;32(8):822.
2. Zeller G, Tap J, Voigt AY, Sunagawa S, Kultima JR, Costea PI, et al. Potential of fecal microbiota for early-stage detection of colorectal cancer. Molecular Systems Biology 2014;10(11).
3. Zhao N, Chen J, Carroll IM, Ringel-Kulka T, Epstein MP, Zhou H, et al. Testing in microbiome-profiling studies with MiRKAT, the microbiome regression-based kernel association test. The American Journal of Human Genetics 2015;96(5):797–807.
