# Supplementary material for: MB-GAN: Microbiome Simulation via Generative Adversarial Network
Source: Gigascience. 2021 Feb 5;10(2):giab005. doi: 10.1093/gigascience/giab005 (PMC7931821; doi:10.1093/gigascience/giab005)
Supplement: giab005_Response_to_Reviewer_Comments_Original_Submission [file giab005_response_to_reviewer_comments_original_submission.pdf]

Author's Response To Reviewer Comments

Close

Dear editor and reviewers,

We have uploaded a PDF file as respond. It has better formats to ease your reading.
Thank you.

Close
