# Supplementary material for: MB-GAN: Microbiome Simulation via Generative Adversarial Network
Source: Gigascience. 2021 Feb 5;10(2):giab005. doi: 10.1093/gigascience/giab005 (PMC7931821; doi:10.1093/gigascience/giab005)
Supplement: giab005_Response_to_Reviewer_Comments_Revision_1 [file giab005_response_to_reviewer_comments_revision_1.pdf]

Author's Response To Reviewer Comments

Close

We would like to thank the editor for allowing us to reformat for publication in GigaScience. We have revised our paper and feel that the new version of the manuscript meets the style of GigaScience.

Comments
Major comment 1: The GigaDB Doi citation needs to be cited in the paper and added to the references. Please see the attached PDF with my marked up comments. If you cited preprints - please check if these are published and update the references accordingly.
Response: We added the GigaDB DOI citation as instructed.
Major comment 2: In addition, please register any new software application in the bio.tools and SciCrunch.org databases to receive RRID (Research Resource Identification Initiative ID) and biotoolsID identifiers, and include these in your manuscript. This will facilitate tracking, repro- ducibility and re-use of your tool.
Response: We added RRID (Research Resource Identification Initiative ID) and biotoolsID iden- tifiers in the manuscript. For your convenience, we have copied the changes below:
....MB-GAN is registered with Research Resource Identification Initiative ID (RRID:SCR 019289) and biotoolsID identifier (biotools:mb-gan). ...

Close
