# Supplementary material for: MB-GAN: Microbiome Simulation via Generative Adversarial Network
Source: Gigascience. 2021 Feb 5;10(2):giab005. doi: 10.1093/gigascience/giab005 (PMC7931821; doi:10.1093/gigascience/giab005)
Supplement: giab005_Reviewer_1_Report_Original_Submission — Karel SedlÃ¡Å™ -- 10/7/2020 Reviewed [file giab005_reviewer_1_report_original_submission.pdf]

Reviewer Report

Title: MB-GAN: Microbiome Simulation via Generative Adversarial Network

Version: Original Submission **Date:** 10/7/2020

Reviewer name: Karel SedlÃ¡Å™

Reviewer Comments to Author:

The manuscript presents revised version of formerly rejected manuscript, I have reviewed earlier this year (as Reviewer #3), dealing with simulation of OTU tables for microbial studies, using generative adversarial network (GAN). Since the authors elaborated the analyses and addressed issues raised during previous review, I believe the manuscript in now worth to be published. Although I do appreciate changes made by authors, I still have some concerns that should be addressed before the manuscript is finally accepted:

1) In response to my former major issue 1 you state "We have accordingly revised the sentence as following: researchers can use MB-GAN to simulate microbiome abundances for a certain sample size and impose the statistical effect sizes on a subset of taxa for different phenotype groups (Zhao et al. 2015)", but in the manuscript the sentence remained in its previous form. Please revise the sentence according to your response, I think it is truly more informative and I got your meaning now.

2) In the response to my former minor issue 5 you state that you have rearranged supplementary figures according to their first referencing in the main text, but this is not true. There is Figure S11 referenced directly after Figure S3, then you reference table S1 which is above Figure S11 in the supplement and so on. So during reading the text, reader have to scroll through the supplement searching for correct items, is it inconvenient and might lead to misunderstandings.

3) On lines 286 - 289 describing Figures S6(a) and S7(a), and in fact any other correlograms in the manuscript you state "A blue ellipse represents a negative correlation, while a red one suggests a positive correlation." However, according to scales next to correlograms, blue represent positive correlations and red represent negative ones. I would rather re-color the figures than change the text as when using red-blue mapping, it is really more common to use red for positive correlations.

4) Please update your GitHub. Actually, I was not able to find revised codes, nor code to perform metaSPARSim simulation. The manuscript should not be published before codes are available.

Methods

Are the methods appropriate to the aims of the study, are they well described, and are necessary controls included? Choose an item.

Conclusions

Are the conclusions adequately supported by the data shown? Choose an item.

Reporting Standards

Does the manuscript adhere to the journal's guidelines on [minimum standards of reporting?] Choose an item.

Choose an item.

Statistics

Are you able to assess all statistics in the manuscript, including the appropriateness of statistical tests used? Choose an item.

Quality of Written English

Please indicate the quality of language in the manuscript: Choose an item.

Declaration of Competing Interests

Please complete a declaration of competing interests, considering the following questions:

- Have you in the past five years received reimbursements, fees, funding, or salary from an organisation that may in any way gain or lose financially from the publication of this manuscript, either now or in the future?
- Do you hold any stocks or shares in an organisation that may in any way gain or lose financially from the publication of this manuscript, either now or in the future?
- Do you hold or are you currently applying for any patents relating to the content of the manuscript?
- Have you received reimbursements, fees, funding, or salary from an organization that holds or has applied for patents relating to the content of the manuscript?
- Do you have any other financial competing interests?
- Do you have any non-financial competing interests in relation to this paper?

If you can answer no to all of the above, write 'I declare that I have no competing interests' below. If your reply is yes to any, please give details below.

I declare that I have no competing interests.

I agree to the open peer review policy of the journal. I understand that my name will be included on my report to the authors and, if the manuscript is accepted for publication, my named report including any attachments I upload will be posted on the website along with the authors' responses. I agree for my report to be made available under an Open Access Creative Commons CC-BY license (http://creativecommons.org/licenses/by/4.0/). I understand that any comments which I do not wish to be included in my named report can be included as confidential comments to the editors, which will not be published.

Choose an item.

To further support our reviewers, we have joined with Publons, where you can gain additional credit to further highlight your hard work (see: https://publons.com/journal/530/gigascience). On publication of this paper, your review will be automatically added to Publons, you can then choose whether or not to claim your Publons credit. I understand this statement.

Yes Choose an item.
